# Maize Intercropping in the Traditional “*Milpa*” System. Physiological, Morphological, and Agronomical Parameters under Induced Warming: Evidence of related Effect of Climate Change in San Luis Potosí (Mexico)

**DOI:** 10.3390/life12101589

**Published:** 2022-10-12

**Authors:** Idrissa Diédhiou, Hugo M. Ramírez-Tobias, Javier Fortanelli-Martinez, Rogelio Flores-Ramírez

**Affiliations:** 1 Facultad de Agronomía y Veterinaria, Universidad Autónoma de San Luis Potosí, Carretera San Luis Potosí-Matehuala Km. 14.5, Soledad de Graciano Sánchez, San Luis Potosí 78321, Mexico; 2 Programa Multidisciplinario de Posgrado en Ciencias Ambientales, Universidad Autónoma de San Luis Potosí. Av. Manuel Nava 201, 2o. piso, Zona Universitaria, San Luis Potosí 78000, Mexico; 3 Instituto de Investigación de Zonas Desérticas, Universidad Autónoma de San Luis Potosí, Calle Altair N° 200, Colonia del Llano, San Luis Potosí 78377, Mexico; 4 Centro de Investigación Aplicada en Ambiente y Salud (CIAAS), CIACYT-Medicina, Universidad Autónoma de San Luis Potosí, San Luis Potosí 78210, Mexico

**Keywords:** climate change, temperatures, heat stress, OTC, intercropping system

## Abstract

Warmer temperatures predicted as a result of climate change will have an impact on *milpa*. An experiment was carried out with induced passive heat with the objective of simulating the increase in temperature on the physiological, morphological, and yield parameters of *milpa* from different climates of San Luis Potosí, Mexico. Two different environments, Open-top chambers (OTC) and control, and three *milpas*, from warm–dry, temperate, and hot and humid climates, were studied. A total of 12 experimental units of 13.13 m^2^ were used in the random design, with a factorial arrangement of 2 × 3 and two replications. Abiotic variables (minimum, maximum, and mean daily temperatures and accumulated heat units) were determined and compared between the two environments and confirmed that the OTC increased the abiotic variables. The growth and development parameters increased under the warming effect. Furthermore, the *milpa* from hot and humid climate was the least affected. In contrast, the warming considerably delayed yield parameters. The squash suffered the most, while the bean benefited the most. The warming affected the chlorophyll fluorescence and gas exchange differently for each crop. However, at an early stage, the maximum photochemical efficiency (Fv/Fm) and non-photochemical quenching (qN) for bean and maize were reduced, while at a late stage, they were Fv/Fm, photochemical quenching (qP), and qN for maize; stomatal conductance and transpiration rate of the squash were improved under the warming treatments. In conclusion, the warming delayed the yield and photosynthetic parameters, while growth and development benefited. The *milpa* systems were differently affected by warming.

## 1. Introduction

*Milpa* is an agroecosystem composed of maize (*Zea mays* L.), bean (*Phaseolus* spp.), squash (C*ucurbita* spp.), and other species that guarantees the foodways’ of Mesoamerica (from central Mexico to the northern and western portions of Central America) [1]. Archaeobotanical and genetic-molecular studies show that maize and *Cucurbita argyrosperma* Hort. Ex L. H. Bayley were domesticated around 9000 BP in western Mesoamerica, as was *Phaseolus vulgaris* L., and being then possible that in this area, they could have been integrated as an agroecosystem [2].

In Mexico, maize is the most important crop, as a large genetic diversity of the crop is reported. This country is also the center of the domestication of *Zea mays*. Mexico’s maize agroecosystems preserve not just germplasm, but also human knowledge and behavioral traditions that reflect the crop’s long co-evolution with human communities [3]. Various management strategies (such as maize intercropping and crop rotation) have evolved in very diverse situations, depending on the climatic, topographic, and biocultural aspects of a specific location [4]. 

This ‘*Milpa*’ system has a number of ecological benefits, including atmospheric nitrogen fixation by *Rhizobium* spp. in symbiosis with bean plants, weed control, soil moisture retention, and erosion protection from the squash. Maize, in turn, offers support for the bean plant as well as shade for the bean and squash. Maize also acts as a physical barrier against illness by preventing the spread of spores [4,5]. The *milpa* system, similar to many others around the world, is based on local technology and the longevity of the plants is dependent on rainfall and climatic conditions [6].

Climate change can cause an increment in the average annual temperature from 1 to 4 °C during the present century in Mexico as in a function of the scenario of population growth [7,8]. Furthermore, it is well is proposed that climate change is affecting the food security of the crops grown under the *milpa* system due to altered environmental conditions such as temperature and an increased frequency of extreme climatic events, creating negative impacts on crop yields [9]. 

Cropping strategies may help to offset the impact of climate change on food security, but few researchers have looked at how temperature rises linked to climate change in agricultural practices affect the *milpa* system plant’s physiological performance [10,11]. Such research is required for a better understanding of regional *milpa* system ecological and functional dynamics [12]. In addition, only a little research work has been undertaken to characterize the physiological performance of plants under various agricultural management systems in the field [13,14]. Most of them focus their efforts on maize, forgetting the complexity of the system with the presence of the other crops (bean and squash). 

There is, therefore, scope for a better understanding of the physiological response of the *milpa* system to the increase in temperatures related to the effects of climate change. The use of an Open-top chamber (OTC) has been one of the most popular methods for simulating potential plant growth and development. The OTC structure has been used by [15,16,17,18] to evaluate the effect of abiotic variables on plants. These studies are helpful in developing strategies for mitigating the negative effects of climate change on plant production in small-scale management systems, where food security is severely challenged by climate change [19].

In the state of San Luis Potosí (SLP) (Mexico), three agroclimatic regions are defined, and in each region, the smallholders are facing an increase in temperature and other problems related to the effect of climate change, such as drought. This way, it is possible to recognize different agroecological regions, from the warm and humid climatic conditions to the dry and hot or temperate ones. In each region, several native genotypes of maize have been reported and are used by the smallholders in their *milpa* systems [20,21,22]. 

Most of the cultivated areas with *milpa* systems in Mexico, and in SLP in particular, are facing critical conditions related to the effect of climate change, such as an increase in temperature. Research, as reported in this research, may provide knowledge on how to contribute to this by investigating the effect of a rising temperature on the *milpa* systems. In addition, this investigation explores this effect using the *milpa* system existing in the state of SLP and evaluates the physiological response from the early to the final stage of each crop in the system. Therefore, the aim of this study was to determine the effect of an increase in temperature on the morphological, physiological, and yield parameters of *milpa* systems from different climates of SLP. The above-mentioned, with the hypothesis that the morphological, physiological, and yield parameters of each *milpa* system and each crop within the system adapted to particular local conditions respond differently and independent of their origins’ climate characteristics when exposed to an increase in temperature, which is related to the effect of climate change.

## 2. Materials and Methods

### 2.1. The Three Milpa System and Their Environments Characteristics

The crops (maize, bean, and squash) were collected in the state of SLP, where three agroecological zones were determined based on mean annual temperature and precipitation, with the average temperatures and precipitation being 14.5, 18.5, and 22.5 °C; 400, 700, and 1200 mm, respectively [20]. These agroecological zones were given the names *Altiplano*, *Media*, and *Huasteca*, and their climates were classified as warm–dry, temperate, and hot and humid, according to [23] adaptations to the Köppen climatic classification system. 

For the selection of the bean and squash, previous experiments (exclusively conducted with maize) in this study allowed us to choose specific farmers for each region. In this context, the farmers who proportionated the maize crops also proportionated the other crops (bean and squash). 

In this sense, generous *Phaseolus vulgaris* beans were collected for farmers from the *Altiplano* and *Media*, while *Vigna unguiculata* (L) Walp bean was collected for farmers from *Huasteca*. It is the most commonly used by the selected *Huasteca* farmers in their *milpa* system. It is important to note that the maize and squash used by the farmers from *Huasteca* are different from the ones used by the farmers of the other regions. However, all the crops used are native genotypes that represent the *milpa* system used by smallholders in each region of the state of San Luis Potosí. 

**Figure 1:**
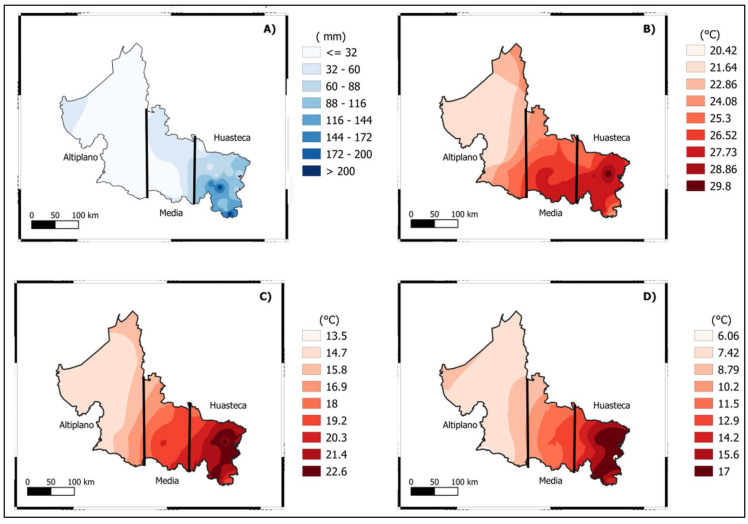
Monthly temperature and precipitation summaries for the state of San Luis Potosí in 2020. (**A**) Precipitation; (**B**) Maximum temperature; (**C**) Mean temperature and (**D**) Minimum temperature. The data were logged from https://smn.conagua.gob.mx/ (accessed on 16th of February 2022).

**Figure 2:**
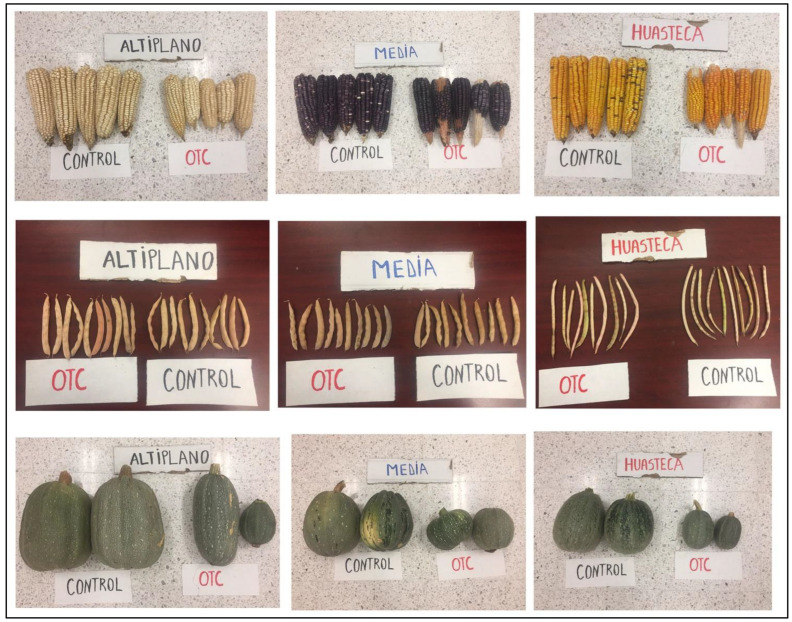
Effect of the induced passive heat on the harvested cobs (maize), pods (bean), and vegetables (squash) of the three *milpa* system. OTC: Open-Top Chamber.

In addition, Figure 1 and Figure 2, and Table S1 describe some features, and all the crops chosen in this study were 3 months old.

### 2.2. Experimental Establishment, Design, and Agronomic Practices

The investigation was carried out at the Faculty of Agronomy and Veterinary of the Autonomous University of SLP. The geographical coordinates of the locality are 100°01′22″ west and 22°12′27″ north, at 1883 m above sea level (m a.s.l.) The geographical area corresponds to the *Altiplano* agroecological zone of the state of SLP, and the climate characteristics are shown in Figure 1 and Table S1.

**Figure 3:**
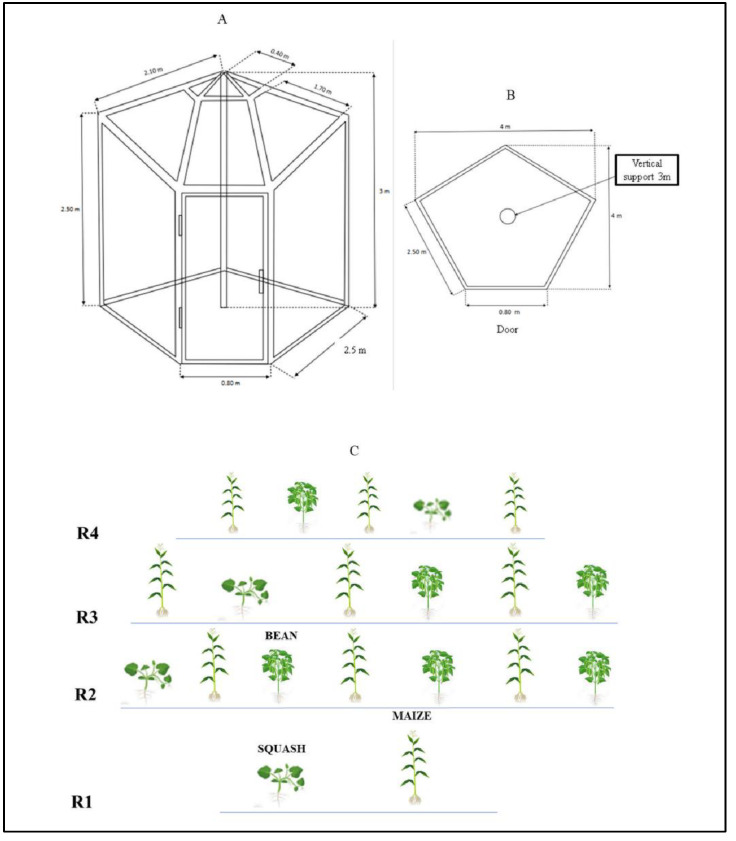
Dimensions and structural details of the open-top chambers (OTC) used to simulate the induced passive heat. (**A**) Frontal view, (**B**) basal view of the OTC, and (**C**) Distribution of the plants (maize–bean–squash) into each plot of controls and OTCs, R: row. In each plot, there were 40 plants of maize (4 in each hole), 12 plants of beans (2 in each hole), and 8 plants of squash (2 in each hole).

The experiment included a total of 12 plots of 13.13 m^2^ (6 plots of Open-Top Chamber (OTC) and 6 plots of control) that resulted in a factorial arrangement of 2 × 3 × 2. The first factor was represented by the environment [passive induced heat with the use of the (OTC) and control], while the last one by the agroecological zone procedence of each *milpa* (*Altiplano*, *Media,* and *Huasteca*). Prior to maize sowing, weeds were manually eliminated from the soil. The maize was sown by hand, placing four seeds in holes at 7 cm in depth along the rows in each environment. In all of the treatments, the maize was sown in June 2021 at an approximate density of 40,000 plants per ha. Bean and squash plants were intercropped with maize plants in a ratio of 2:1 in each block, respectively, for a total of 8 plants of squash and 12 plants of bean in each block (Figure 3). The bean seeds and squash plants were sown and planted 30 days after the maize to avoid competition between the seedlings [12]. Agronomic practices and plant protection measures (daily irrigation to prevent the effect of drought and elimination of undesirable plants) were accomplished throughout the crop’s growth period. Irrigation was undertaken immediately after sowing.

### 2.3. Simulation of the Induced Passive Heat

Open-top chamber (OTC) structures were used to simulate the induced passive heat. These structures allow for passive heating and are a simple method for monitoring plant responses to abiotic variables such as temperature increases in the field [16,17,24,25]. The finished structures were pentagonal at the surface base, with a perimeter of 10.8 m [(2.5 m × 4) + 0.8 m] and a height of 3 m (Figure 3A,B). Each OTC was covered with transparent natural tubular plastic. When compared to external ambient circumstances, this OTC design raises the air temperature. Across the experiment, the magnitude with which OTCs altered the microclimate (air temperature) was regularly recorded both within and outside these structures.

### 2.4. Abiotic Variables Measurement 

The temperatures were registered with data-loggers HOBO U23 (Onset Computer Corporation, Bourne, MA, USA). In two selected OTC and control plots, two data loggers were mounted 15 cm and 150 cm above the ground in the center. These two positions allow us to monitor the air temperature in the relative space where the three crops are established. The readings were scheduled to be taken every hour and averaged daily. These measurements were taken from 27 June to 12 November 2021, and the daily mean, minimum, and maximum air temperatures in each environment were calculated using the recorded data. With the daily mean air temperature, the daily accumulated heat units were calculated with the residual classic method, which uses the following expression [26].

Daily accumulated heat units = DMAT−Tb

where:

DMAT: Daily mean air temperature

Tb: base temperature

The daily accumulated heat units for maize were calculated with the data logged at 150 cm with a 10 °C base temperature [27], while the crops (bean and squash) were calculated with the logged data at 15 cm with a base temperature of 8.3 °C. In addition, the sums of the daily accumulated heat units during all of the experiments were used to determine the accumulated heat units or growing degree days (GDD) for each environment and were compared between the two treatments.

### 2.5. Morphological, Physiological, Yield and Yield Components Variables Measurement

Morphological, physiological, yield and yield component variables were determined for each crop in the intercropping system of the *milpa*. Table S2 summarizes the variables’ measurements.

#### 2.5.1. Morphological Variables Measurement 

The rate of growth, plant height, stem thickness, leaf length, the width of leaf, leaf area, height to ear insertion, days to female and male flowering, number of flowers, and leaves were used to determine the growth and development dynamic of the crops in each plot (Table S2).

The rate of growth (RG) was defined as the increment in the longitude of the plants measured from the base of the soil to the top of the plant height. The RG for maize was determined from 30 days after the first emergence to 170 days in m day^−1^. For bean and squash, they were determined from 30 days after the first emergence to 135 days in cm day^−1^. The following formula was used: 

The equation below was used:

$$\mathrm{RG}\;=\frac{\mathrm{PH}2-\mathrm{PH}1}{T2-T1}$$

where: PH1 and PH2 are the plant height, T1 and T2 the previously indicated times.

The plant height was measured from the ground surface to the tip of the plant. The stem diameter (mm) was measured using the Vernier Caliper; it was measured at 10 cm above the ground level for each crop. The leaf characteristics (length and width) were evaluated in three leaves (one above and two below the leaf associated with the ear). The general equation was used to estimate the individual leaf area of maize [28]:

Leaf area = L ×W ×A 

where L and W are the length and width, respectively, of the leaf. The height of the ear insertion was measured from the distance between the ground surface and the ear insertion of the selected maize. The male and female flowering were measured on each plot. Male flowering was recorded as the number of days from sowing to the first anther extrusion. Female flowering was the number of days from sowing to the first visible silk. The number of flowers and leaves per plant was estimated by counting the number of flowers and leaves on the bean and squash plants; in the case of maize, only the number of leaves was determined.

#### 2.5.2. Physiological Variables Measurement

##### Measurement of Plant Chlorophyll Fluorescence Parameters

On fully open leaves, the chlorophyll fluorescence variables were measured using a portable photosynthesis system (LI-6400XT, LI-COR Biosciences, Lincoln, USA) fitted with a fluorescence chamber (LI-6400-XT). At predetermined intervals, minimal (Fo) and maximal (Fm) fluorescence were measured, followed by a 0.2 s weak modulated saturating light flash. Actinic light at 1600 mol m^−2^ s^−1^ was used to illuminate the leaf. It followed the application of saturating light pulse for 0.8 s to record Fm. The actinic light was switched off, and far-red light was applied to determine Fo. The total energy harvesting efficiency in the light, NPQ (alternative non-photochemical quenching) and qN (non-photochemical quenching), and electron transport rate (ETR) were calculated. The following equation was used to compute the photochemical quenching (qP) parameters, the proportion of open PSII, and the quantum yield of PSII (PhiPS2). The following equations were reported from [29,30,31]:

$$\frac{\mathrm{Fv}}{\mathrm{Fm}}=\frac{\mathrm{Fm}-\mathrm{Fo}}{\mathrm{Fm}}$$

$$\mathrm{qP}\;=\frac{F´m-\mathrm{Fs}}{F´m-F´o}$$

$$\mathrm{PhiPS}2=\frac{F´m-\mathrm{Fs}}{F´m}$$

$$\mathrm{NPQ}\;=\left(\frac{\mathrm{Fm}}{F´m}\right)-1$$

ETR = PhiPS2.PPFD.α.β

where:

Fv/Fm: Maximum efficiency of the Photosystem II (PSII)

Fo: Basal chlorophyll a fluorescence (in the dark) Minimal F (Fluorescence signal (zero subtracted))

F´o: Basal chlorophyll a fluorescence (after light–dark transition) Minimal F, light adapted

Fm: Maximum chlorophyll a fluorescence, dark adapted

F´m: Maximum chlorophyll a fluorescence, light adapted

Fs: Apparent chlorophyll a fluorescence in the light-adapted steady-state fluorescence

PPFD: Photosynthetic photon flux density

α denotes the leaf absorbance, and β is the partitioning of the absorbed quanta between photosystems I and II. The latter was assumed to be 0.5, indicating that an equal distribution of excitation energy occurs between two photosystems, while the former is assumed as 0.86 [32].

##### Plant Gas Exchange Parameters Measurement

The parameters of gas exchange were analyzed to understand more about the plant physiology and photosynthetic machinery of the crops from different climates under the effect of passive induced heat. A fully sun-exposed state was used to record the leaf gas exchange parameters: CO_2_ assimilation photosynthetic rate (µmol CO_2_ m^−2^ s^−1^)), stomatal conductance (mmol H_2_O m^−2^ s^−1^), transpiration rates (mmol H_2_O m^−2^ s^−1^) and the intrinsic water-use efficiency (iWUE (µmol CO_2_ mol^−1^ H_2_O) as the relationship between photosynthetic rate and transpiration [33,34]. Additionally, with a portable photosynthesis system (LI-6400XT, LI-COR Biosciences, Lincoln, USA), the youngest fully developed leaves were used, and the measurements were recorded from an intermediate leaf position on one side of the central nerve for maize genotypes [34]; while for bean and squash, competitive plants were selected and the intermediate leaves were used for the measurements. 

Prior to measurements of chlorophyll fluorescence and gas exchange parameters in light conditions, photosynthetic active radiation (PAR) was monitored near the plants with the PAR sensor of the LI-6400XT chamber [30,35]; and the values were estimated at 1500 μmol m^−2^ s^−1^ in the control plots and 800 μmol m^−2^ s^−1^ in OTC plots for maize genotypes, while for bean and squash, the values were 100 and 180 μmol m^−2^ s^−1^, respectively, due to the shade under maize plants. The level of PAR was provided for measuring leaves as actinic light (10% blue light and 90% red light) passed the LI-6400XT leaf chamber during the assessment. The photosynthetic parameters were obtained directly (except iWUE) from the portable photosynthesis system LI 6400XT, and its calculation was established on the LI-6400XT instruction manual (LI-6400 T Instruction Manual, v6, LI-COR Biosciences, Inc. Lincoln, USA). The measurements were taken at 45 and 75 days after the emergence of the crops, corresponding to the early stage and physiological maturity of the crops, respectively.

#### 2.5.3. Yield Variables and Components Plants

Different yield variables and components were determined for the three crops for each *milpa* system. For maize, cob diameter (mm), cob weight (g), cob length (cm), number of rows per cob, number of cobs per plant, number of grains per row, 100 grains weight per plot (g), and yield (t ha^−1^) were registered. Ten cobs were used to determine the mentioned variables, while the number of cobs per plant was measured on 10 plants in each plot. However, for bean and squash, only the yield (t ha^−1^) parameter was determined for each crop.

### 2.6. Statistical Analysis

The data for the morphological, physiological, and yield variables were analyzed using the GLM procedure of the Statistical Analysis System (SAS, 2003) program. The model is characterized by two fixed factors, namely ‘genotypes’ and ‘environment’, as well as their interaction ‘genotypes x environment’ for each crop. The Tukey test was used to check for significant differences between the treatment means. If *p* < 0.05, the effects and interactions were considered significant. The data were examined for normality before being analyzed, and transformation was employed to correct them. The abiotic variables were analyzed using a repeated measure analysis of variance (ANOVA). They were compared between the OTC and control environments and summarized for each data-logger. The data shown are the means and standard error. The vertical bars signify the standard error, and, on the top, different letters represent the significant differences among the means according to Tukey’s test (*p* < 0.05). The correlations between the abiotic variables and the morphological, physiological, and yield parameters were conducted in the Paleontological Statistics Software package for education and data analysis (Past 4.0).

## 3. Results

### 3.1. Abiotic Variables under OTC and Control Plots

**Figure 4:**
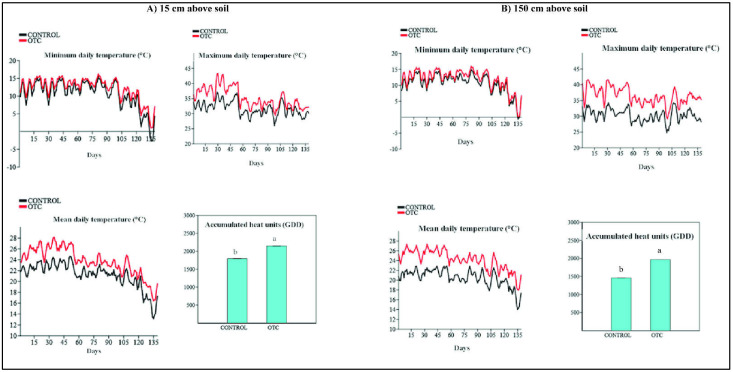
Average daily values of registered temperatures and accumulated heat units calculated in the Open-Top Chamber (OTC) and in the control environments at (**A**) 15 cm and (**B**) 150 cm above the soil. Vertical bars indicate the standard error for the accumulated heat units during all the experiments (*n* = 2). Different letters represent significant difference among the means according to Tukey’s test (*p* < 0.05).

Overall, the minimal, maximal, and mean daily temperatures all increased significantly in the OTC treatments, and a significant difference was also recorded for the accumulated heat units at the two evaluated positions (Figure 4).

During the experiment, at 15 cm above the soil, the minimal daily temperature (mean ± error standard) was 10.68 ± 0.37 °C in the control plots and 12.17 ± 0.3 °C in the OTC plots. This variable significantly differed between the environments (Fvalue = 2.29, CM = 152.68, and *p* = 0.0025), and that means the structure of OTC increased by an average of 1.49 °C, the minimum daily temperature during the experiment. The maximum daily temperature was 35.26 ± 0.31 °C inside the OTC and 31.7 ± 0.23 °C within control and significantly differed between the two environments (Fvalue = 82.05, CM = 880.24, and *p* < 0.0001), and the use of OTC increased up to 3.56 °C in comparison to the control. The mean daily temperature was 21.20 ± 0.22 °C in control plots and 23 ± 0.23 °C in OTC plots. This variable significantly differed between the treatments (Fvalue = 82.05, CM = 880.24, and *p* < 0.0001), and that means the structure of OTC increased the mean daily temperature during the experiment to 1.8 °C. Then, the accumulated heat units recorded in OTC were statistically superior to the ones inside the control plots. The OTC recorded 350.36 GDD (Growing Degree Days) more in comparison to the control during the 139 days. That means the induced passive heat increased the accumulated heat units during all the experimentation (Figure 4A).

At 150 cm above the soil, the minimal daily temperature was 10.65 ± 0.29 °C in control and 11.87 °C in OTC plots. The difference between the two conditions was significant (Fvalue = 7.82; CM = 103.65 and *p* = 0.005), indicating that the OTC raised the minimum daily temperature up to 1.22 °C. The maximum daily temperature was 36.38 ± 0.27 °C inside the OTC and 30.30 ± 0.22 °C within control and significantly differed between the two environments (Fvalue = 298.5, CM = 2565.89 and *p* < 0.0001), and the use of OTC increased up to 6.08 °C in comparison to the control environment. The mean daily temperature was 20.48 ± 0.17 °C in the control plots and 24.13 ± 0.19 °C in the OTC plots. This variable showed significant differences between the environments (Fvalue = 195.32, CM = 925.25 and *p* < 0.0001), showing that the OTC structure increased the mean daily temperature by 3.65 °C during the experiment. A total of 1964.17 ± 13.97 GDD was recorded in the OTC plots vs. 1459 ± 10.37 GDD in the control plots during the duration of the experiment. A significant difference was observed between the two environments (Fvalue = 195.32, CM = 925.25 and *p* < 0.0001). 504.18 GGD more was obtained in the OTC plots in comparison to the control plots (Figure 4B).

### 3.2. Effect of the Induced Passive Heat on Milpa Morphological Variables

Significant effects of the genotypes and the environment on some of the physiological variables using analysis of variance were observed. The interactions Environment (E) × Genotypes (G) (E × G) were significant for the plant height, width of leaf, and rate of growth for maize; the number of leaves per plant and stem thickness for bean; and the number of leaves per plant, stem thickness, plant height, and rate of growth were significant for squash (Table S3). When the interaction was not significant, the simple effect of the environmental and genotypic factors was considered.

As seen in Table 1, the mentioned interactions (E × G) respond to the growth and development variables of each crop from each agroecological zone at each treatment. 

The passive induced heat increased the plant height and rate of growth of the maize genotypes. The maize genotypes in the OTC plots reached a mean of 2.57 ± 0.09 m for plant height, while in the control plots, it was 2.06 ± 0.16 m; and 0.013 ± 0.0008 m day^−1^ in OTC plots and 0.010 ± 0.001 m day^−1^ in control plots. The genotypes from *Huasteca* (hot and humid climate) in the OTC and control plots showed the maximum plant height and rate of growth and were significantly superior to those from warm–dry (*Altiplano*) and temperate (*Media*), where the induced passive heat did not affect the plant height and rate of growth of the maize genotypes. The induced passive heat affected the width leaf of the maize, where the genotypes grown in control plots showed a mean of 11.17 ± 0.06 cm vs. 9.05 ± 0.44 cm in OTC plots. However, the width of the leaf was statistically equal for the maize in control plots and superior to the ones reported in OTC for each genotype. Under control and OTC conditions, the E × G interaction for stem thickness reported no difference for the maize plants. That mean, the stem thickness reported under the two conditions was statistically equal for each genotype. However, more stem thickness was registered under control for genotypes from *Media* (temperate climate) and *Altiplano* (warm–dry climate), while for *Huasteca* (hot and humid climate), the maximum stem thickness was observed under OTC plots. 

The number of leaves per plant decreased significantly under the OTC for the E × G of the squash plants, wherein in the control environment, the mean was 60.5 ± 1.42 vs. 35.38 ± 4.5 under the OTC plots. That means the induced passive heat decreased by 41.52% the number of leaves per plant of the squash. The genotypes from the temperate climate (*Media*) were the most affected, with a significant difference under the induced passive heat. Under the E × G, the plant height and rate of growth were affected by the induced passive heat of the squash. The squash in the control plots registered a plant height and rate of growth of 106.72 ± 3.77 cm and 0.7 ± 0.06 cm day^−1^, respectively, against 97.92 ± 0.56 cm and 0.6 ± 0.00001 cm day^−1^ under OTC conditions. The genotypes from *Huasteca* (hot and humid climate) and *Altiplano* (warm–dry) reported significant differences in plant height and rate of growth, while the ones from *Media* (temperate climate) showed no difference. Additionally, for the stem thickness, only the genotypes from the hot and humid climate (*Huasteca*) registered significant differences under the effect of the passive heat, while for the temperate (*Media*) and warm–dry (*Altiplano*), no differences were observed. 

For bean, the E × G revealed no differences. However, the bean from *Huasteca* (hot and humid climate) registered a significant difference in stem thickness in comparison to the ones from warm–dry and temperate climates (*Altiplano* and *Media*, respectively). A mean of 10.79 ± 0.51 mm and 9.45 ± 0.54 mm was observed for the beans from *Huasteca* grown under OTC and control environments, respectively. On the other hand, the means were 5.55 ± 0.25 mm (OTC) vs. 6.33 ± 0.35 mm (control) and 6.32 ± 0.16 mm (OTC) vs. 6.74 ± 0.22 mm (control) for the beans from warm–dry (*Altiplano*) and temperate (*Media*) climates, respectively. Finally, the number of leaves per plant was affected by the induced passive heat. The E × G showed a significant difference for the beans from *Huasteca* (hot and humid climate) and *Altiplano* (warm–dry climate), where the number of leaves per plant for the bean grown under control was statistically superior to the ones under passive heat conditions, while no difference was observed between bean from *Media* (temperate climate). Overall, 34.04% more leaves were registered under control conditions for the beans from *Huasteca* (hot and humid climate) in comparison to OTC conditions, while for *Altiplano* (warm–dry climate), the effect of the passive heat decreased the number of leaves by 42.97% (Table 1).

Simple effects of the factors (Environment and Genotypes) were observed for leaf number per plant, leaf length, leaf area, days for female flowering per plot, days for male flowering per plot and height to ear insertion for maize; the number of flowers per plant, plant height and rate of growth for bean and number of flowers per plant for squash (Table S3).

Table 2 shows the simple effect of passive heat and controlled environments over variables of growth and development for maize, bean, and squash plants. For the factor environment, the OTC decreased significantly the leaf number per plant, leaf area, days for male flowering per plot and height to ear insertion for maize plants, number of flowers per plant, and rate of growth for beans, and finally, the number of flowers per plant for squash. 

No differences were observed for the variables leaf length and days for female flowering per plot for maize and plant height for beans. The OTC decreased the leaf area of the maize up to 132.64 cm^2^, but it significantly accelerated the days for male flowering per plot to 6.2 days and the height to ear insertion to a mean of 1.41 ± 0.03 m vs. 0.98 ± 0.03 m in the control conditions. For beans, the number of flowers per plant and the rate of growth were affected by the passive heat and decreased by up to 7.3 and 0.05 cm day^−1^, respectively. The squash was one of the crops most affected by the induced passive heat for the variables number of flowers per plant, where it decreased by up to 61.97% in comparison to the control environments. 

For the factor genotypes represented by the climate procedence of the crops, the genotypes from the hot and humid climate (*Huasteca*) registered a significant difference and were statistically superior to the ones from the warm–dry and temperate climates (*Altiplano* and *Media*, respectively) for the variables leaf number per plant, leaf length, leaf area, days for female flowering per plot, days for male flowering per plot, and height to ear insertion for maize plants. The genotypes from *Huasteca* (hot and humid climate) registered 103.9 ± 1.35 cm of leaf length, while the ones from *Altiplano* (warm–dry) and *Media* (temperate) were 93.0 ± 0.3 and 90.4 ± 0.2 cm, respectively. In addition, more leaf area was observed in the maize from *Huasteca* (hot and humid climate) with 810.29 ± 20.9 cm^2^, while for genotypes from *Altiplano* (warm–dry), it was 723.12 ± 16.9 cm^2^. The genotypes from *Media* (temperate) registered the least leaf area. On the other hand, the genotypes from *Huasteca* (hot and humid climate) took more time to reach the female and male flowering stages, with a mean of 67.2 ± 1.51 days and 66.2 ± 1.51 days, respectively, while the maize from *Altiplano* (warm–dry) and *Media* (temperate) took less time to reach their reproductive stage. 

In addition, for the number of flowers per plant, the bean from the hot and humid and temperate climates (*Huasteca* and *Media*, respectively) registered the maximum flowers, while for the squash, no differences were observed between the three climates. For plant height, the beans from hot and humid and temperate climates (*Huasteca* and *Media*, respectively) showed the maximum values, while for the rate of growth, it was the hot and humid and warm–dry climates (*Huasteca* and *Altiplano*, respectively) where the maximum values were registered with a mean of 0.16 ± 0.01 and 0.13 ± 0.01 cm day^−1^, respectively (Table 2). 

### 3.3. Effect of the Induced Passive Heat on the Yield and Yield Components on Milpa 

Significant effects of the genotypes and the environment over some of the yield and its component variables using analysis of variance were observed. The interactions Environment (E) × Genotypes (G) (E × G) were significant for the cob diameter, cob weight, and the number of rows per cob for maize; yield for squash and bean (Table S4). When the interaction was not significant, the simple effect of the environmental and genotype factors was considered.

**Figure 5:**
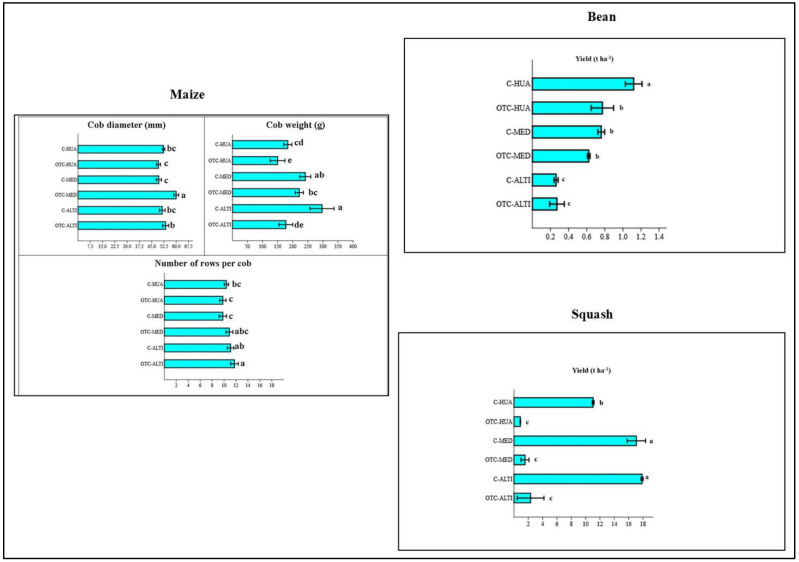
Effect of induced passive heating on cob diameter, cob weight, number of rows per cob for maize plants; yield for squash and bean from different climates of the state of San Luis Potosí (Mexico). OTC: Open-top Chamber; C: Control; HUA: *Huasteca* (hot and humid climate); MED: *Media* (temperate climate); ALTI: *Altiplano* (warm–dry climate). The letters a, b, c, d, and e indicate significant differences according to the Tukey test (*p* < 0.05). The values are the means ± SE (standard error). The environment × genotype interaction was significant for all the parameters according to the Tukey test (*p* < 0.05).

Figure 5 depicts the yield and its component variables’ responses to the *milpa* system as a result of the combined effect of the factors environment and genotype. 

The yield components of the maize showed different responses to the combination of different environments and genotypes for the cob diameter, the cobs from the temperate climate (*Media*) registered the maximum values and were statistically superior to the ones from warm–dry and hot and humid climates (*Altiplano* and *Huasteca*, respectively). That means the passive induced heat benefited the cob diameter of the maize from the temperate climate (*Media*), while for the two last ones, there were no differences in cob diameters between the OTC and control treatments. The maximum values of the cob weight were registered in the interaction control environment and the maize genotypes. Under this interaction, the cob weight was 297.32 ± 19.87 g, 241.74 ± 9.01 g, and 183.67 ± 6.65 g for *Altiplano* (warm–dry), *Media* (temperate), and *Huasteca* (hot and humid), respectively. That means the induced passive decreased the cob weight, and the minimum values were recorded for genotypes from hot and humid and warm–dry climates (*Huasteca* and *Altiplano*, respectively) (Figure 5). 

The maximum number of rows per cob was recorded under the interaction OTC in cobs from warm–dry climate (*Altiplano*) with 11.75 ± 0.31 and 11.1 ± 0.27 under control conditions, and there was no statistical difference between them. Additionally, no statistical differences were observed for the number of rows per cob of the genotypes from *Huasteca* and *Media*. That means the induced passive heat did not affect the number of rows per cob of the maize. 

**Figure 6:**
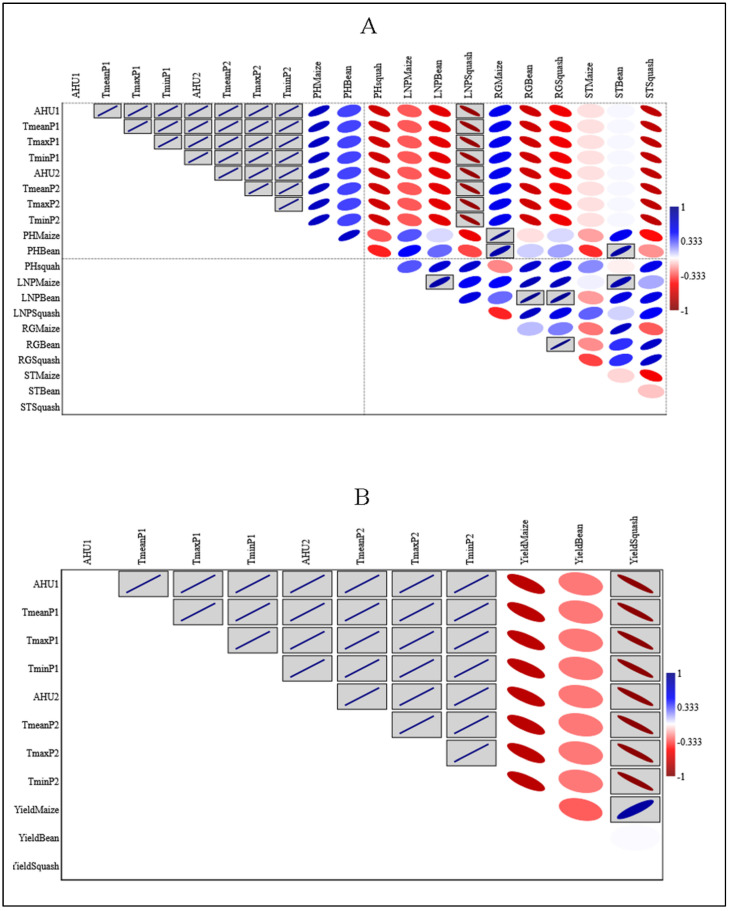
Plots of the statistic correlation of Pearson linear (r) among the abiotic variables and morphological and yield parameters of the milpa. AHU: accumulated heat units; Tmean: mean daily temperature; Tmax: Maximum daily temperature; Tmin: minimum daily temperature; P1: at 15 cm and P2: at 150 cm above the soil; PH: plant height; LNP: leaves number per plant; RG: rate of growth; ST: stem thickness. (**A**): Correlation among morphological and abiotic variables and (**B**): Correlation among yield of each crop of the *milpa* system and abiotic variables. The boxed plots are significant at *p* < 0.05.

The squash yield was one of the most affected by the induced passive heat (Figure 2 and Figure 5). A significant difference was recorded in the E × G where, under OTC plots, the yield decreased in comparison to control plot values. The maximum yield was registered under control plots from *Altiplano* (warm–dry) and *Media* (temperate). The induced passive heat decreased the yield of the squash by up to 87.02% and 90.92% in the warm–dry (*Altiplano*) and temperate climates (*Media*), respectively (Figure 6). Additionally, for the squash from *Huasteca* (hot and humid), a loss of yield was observed, with a value of 91.94% in comparison to control plots. 

For the beans, the yield was affected significantly and decreased under the effects of the passive heat for the genotypes from the hot and humid climate (*Huasteca*) with 1.12 ± 0.03 t ha^−1^ in control plots vs. 0.77 ± 0.04 t ha^−1^ in OTC conditions. On the other hand, no statistical differences were recorded for the genotypes from *Media* and *Altiplano* (temperate and warm–dry climates, respectively) under the OTC and control environments. However, the bean from *Altiplano* registered the lowest yield under OTC and control environments (Figure 5).

Simple effects of the factors (Environment and Genotypes) were observed for a number of cob per plant (NCP), cob length (CL), number of grains per row (NGR), 100 grains weight per plot (100 GW), and yield (Y) for maize (Table S4).

Table 3 shows the simple effect of maize yield and its component variables under the effect of passive heat and control environments. Under the environmental factor, induced passive heat significantly reduced all yield and its component variables for maize.

The number of cobs per plant, cob length, the number of grains per row, 100 grains weight per plot, and yield decreased by up to 0.94, 3.95 cm, 6.95, 6.77 g, and 2.33 t ha^−1^, respectively, in comparison to the control conditions.

Under the factor genotypes represented by the climate procedence of the maize, the genotypes from the hot and humid climate (*Huasteca*) showed significant differences in the number of cob per plant and number of grains per row and were statistically superior to those registered from *Altiplano* and *Media* (warm–dry and temperate climates, respectively), while for cob length no differences were registered for the factor genotype of the crops. In addition, the genotypes from *Altiplano* and *Media* (warm–dry and temperate climates, respectively) registered the maximum values of 100 grains weight per plot and yield, with means of 48.39 ± 2.14 g and 49.55 ± 0.43 g and 5.08 ± 0.73 t ha^−1^ and 4.62 ± 0.48 t ha^−1^ yields for *Altiplano* and *Media* (warm–dry and temperate climates, respectively) while from *Huasteca* (hot and humid); the values were 38.84 ± 2.7 g (for 100 grains weight per) and 2.93 ± 0.61 t ha^−1^ (for yield), being the one with the least yield and 100 grains weight per plot for maize genotypes (Table 3).

### 3.4. Effect of Induced Passive Heat on Photosynthetic Capacity of Milpa System 

#### 3.4.1. Effect of Induced Passive Heat on Chlorophyll Fluorescence Parameters Measured at 45 Days after Emergence of Each Crop of the Milpa 

Significant effects of the genotypes and the environment on some of the chlorophyll fluorescence parameters using analysis of variance were observed. For the interactions Environment (E) × Genotypes (G) (E × G), Electron Transport Rate (ETR), Alternative non-photochemical quenching (NPQ), Quantum yield of the Photosystem II (PhiPS2), Non-photochemical quenching (qN), and Photochemical quenching (qP) were significant for beans and squash (except NPQ), but none of the parameters were significant for the interaction E × G for maize (Table S5). When the interaction was not significant, the simple effect of the environmental and genotypic factors was considered.

Table 4 shows the chlorophyll fluorescence parameters measured in each crop 45 days after emergence. 

No differences were recorded for the effect of the induced passive heat on ETR with 58.2 ± 3.9 μmol m^−2^ s^−1^ in OTC plots and 61.1 ± 3.3 μmol m^−2^ s^−1^ in control plots for maize genotypes. However, under the genotype effect, the maize from *Huasteca* (hot and humid climate) showed the maximum ETR (71.7 ± 5.2 μmol m^−2^ s^−1^) and was statistically superior to those from warm–dry (*Altiplano*) and temperate climates (*Media*) with 54.2 ± 3.3 and 53.1 ± 3.5 μmol m^−2^ s^−1^, respectively.

ETR was found to be higher in OTC plots from hot and humid climates (*Huasteca*), with a mean of 51.4 ± 0.5 μmol m^−2^ s^−1^ and 41.4 ± 1.01 μmol m^−2^ s^−1^ in the control plots, which were statistically superior to the values recorded in the E × G from *Altiplano* and *Media* (warm–dry and temperate climates, respectively), where the lowest values were recorded in the beans from *Altiplano*, with 15.2 ± 1.7 μmol m^−2^ s^−1^ in OTC and 12.3 ± 0.5 μmol m^−2^ s^−1^ in the control plots. That means that the induced passive heat increased the ETR of the bean from the hot and humid climates (*Huasteca*). 

On the other side, the ETR of the squash (from *Media* and *Huasteca*) was significantly impacted by the produced passive heat with a significant difference. The higher values were reported under the control plots from *Media* and *Huasteca* (temperate and hot and humid climates, respectively), with values of 81.03 ± 1.29 and 62.13 ± 1.14 μmol m^−2^ s^−1^, respectively. The induced passive heat decreased by up to 38.36 and 41.74 μmol m^−2^ s^−1^ in comparison to control plots from *Media* (temperate) and *Huasteca* (hot and humid), respectively. The squash from *Altiplano*, on the other hand, benefited from the influence of the produced passive heat and was significantly higher than the squash planted in the control plots. 

The most important quenching parameters in assessing plant performance under stress circumstances are Fv/Fm, PhiPS2, and qP. In this approach, the produced passive heat harmed the bean and squash crops, lowering their Fv/Fm, which indicates photosystem II’s maximal photochemical efficiency and potential activity in plant leaves. 

For bean and squash, the decreases were 0.07 and 0.05, respectively. However, for maize, the passive heat increased the Fv/Fm with significant differences, and the values were 0.75 ± 0.008 in OTC and 0.72 ± 0.005 in control plots. Additionally, for PhiPS2, the maximum value was reported in OTC with 0.08 ± 0.006 and 0.05 ± 0.005 under the control conditions for maize genotypes. That indicates that the position of the leaves inside the plots can influence the chlorophyll fluorescence parameters. Under the genotype factor, the maize from hot and humid (*Huasteca*) reported a significant difference, and the mean was 0.08 ± 0.008, while for the others from *Altiplano* (warm–dry) and *Media* (temperate), the mean was 0.06 ± 0.008 for each. 

The E × G indicated that the maximum value of PhiPS2 in the beans was recorded in the control plots from *Altiplano* (warm–dry) and *Media* (temperate), with 0.26 ± 0.01 vs. 0.15 ± 0.01 in OTC and 0.07 ± 0.001 in control vs. 0.05 ± 0.001 in OTC, respectively. In addition, for the beans from *Huasteca* (hot and humid), no difference was reported between control and OTC. For squash, the maximum PhiPS2 was recorded in control plants from *Altiplano* (warm–dry), but the differences were not significant for each genotype in the two environments. 

Non-photochemical quenching (qN) and alternative non-photochemical quenching (NPQ) were impacted by the effect of the induced passive heat, where their values under the control conditions were significantly higher than that reported in OTC for the maize genotypes. qN and NPQ in control were 0.91 ± 0.003 and 1.84 ± 0.04 while in OTC were 0.84 ± 0.01 and 1.55 ± 0.06, respectively. 

For the beans, the E × G reported the maximum values of qN in the control conditions from *Huasteca* (hot and humid), but the difference was not significant for the two conditions. On the other hand, the induced passive heat significantly affected the bean from *Altiplano* and *Media* (warm–dry and temperate climates, respectively); they decreased by up to 24.24% and 50%, respectively, the qN in comparison to the control conditions. 

In comparison to control conditions, the induced passive heat impacted the squash from *Media* (temperate), where they lost up to 62.07% of the qN. However, the induced passive heat significantly increased the qN of the squash from *Altiplano* (warm–dry), where the mean was 0.33 ± 0.07 in OTC and 0.16 ± 0.04 in control, while no difference was reported for the squash from *Huasteca* (hot and humid). That means that the procedence of the plants may influence the qN parameters. 

The qP reported no difference for the maize genotypes under the effect of the passive heat, while for the factor genotype, the maize from a hot and humid climate (*Huasteca*) showed maximum values of 0.37 ± 0.02 and was statistically superior to the qP of the genotypes from *Altiplano* and *Media* (warm–dry and temperate climates, respectively).

For the beans, the E × G reported a significant difference for the plants from *Media* (temperate), where the induced passive heat increased the qP of the bean by 0.55 ± 0.01 vs. 0.31 ± 0.06 in the control environment. On the other hand, no statistical differences were recorded for the bean from *Altiplano* and *Huasteca* (warm–dry and hot and humid climates, respectively) under the effect of the passive heat; however, the ones from *Huasteca* reported up to 0.23 qP in comparison to the OTC conditions. 

The qP of the squash increased significantly under the OTC conditions for the plants from a hot and humid climate (*Huasteca*), where the qP increased by up to 57.14% in comparison to control conditions, while no differences were reported for the squash from *Altiplano* and *Media* (warm–dry and temperate climates, respectively) under the effect of the passive heat (Table 4).

#### 3.4.2. Effect of Induced Passive Heat on Gas Exchange Parameters Measured at 45 Days after Emergence of Each Crop of the Milpa 

Significant effects of the genotypes and the environment on the gas exchange parameters using analysis of variance were observed. For the interactions, Environment × Genotypes (G) (E × G), stomatal conductance (Cond), intrinsic water-use efficiency (iWUE), photosynthetic rate (Photo), and transpiration rates (Trmmol) were significant for bean and squash (except iWUE and Photo), but none of the parameters were significant for maize (Table S6). When the interaction was not significant, the simple effect of the environmental and genotypic factors was considered.

Table 5 shows the gas exchange parameters under the effect of the passive heat on the *milpa* system 45 days after emergence from different climates. The passive heat significantly affected the CO_2_ assimilation, also known as the photosynthetic rate (Photo) of the maize, with a decrease of 8.25 µmol CO_2_ m^−2^ s^−1^. For the factor genotype, the maize from a hot and humid climate (*Huasteca*) showed the maximum values of CO_2_ assimilation with a mean of 39.15 ± 2.61 µmol CO_2_ m^−2^ s^−1^ and was statistically superior to the reported from *Altiplano* and *Media* (warm–dry and temperate climates, respectively). 

On the other hand, the E × G revealed different responses for bean and squash. For the two crops, the induced passive heat increased the photosynthetic rate with maximum values of 52.2 ± 1.18 and 63.89 ± 1.56 µmol CO_2_ m^−2^ s^−1^ for *Huasteca* (hot and humid climate) in OTC conditions for bean and squash, respectively. In addition, the induced passive heat significantly reduced the photosynthetic rate of the bean from temperate climates (*Media*), where the values in the control plots (42.96 ± 0.93 µmol CO_2_ m^−2^ s^−1^) were significantly superior to the mean in the OTC conditions (29.9 ± 3.89 µmol CO_2_ m^−2^ s^−1^) while no differences were recorded for the squash from warm–dry and temperate climates (*Altiplano* and *Media*, respectively) and for bean from warm–dry climate (*Altiplano*).

The induced passive heat increased the stomatal conductance (Cond) of the maize genotypes. A significant difference of up to 42.31% in the stomatal conductance in comparison to control conditions. Additionally, the maize from hot and humid climates (*Huasteca*) reported the maximum value of stomatal conductance with 0.25 ± 0.02 mmol H_2_O m^−2^ s^−1^, which was statistically superior to the other maize. 

The E × G revealed the different responses of the bean, where maximum values were reported for the plants from *Huasteca* (hot and humid climate) with 0.62 ± 0.03 mmol H_2_O m^−2^ s^−1^ in OTC plots vs. 0.55 ± 0.05 mmol H_2_O m^−2^ s^−1^ in the control conditions, which were statistically equal. In addition, the bean from the warm–dry climate (*Altiplano*) significantly increased the stomatal conductance under the effect of passive heat with 0.4 ± 0.03 mmol H_2_O m^−2^ s^−1^ and 0.22 ± 0.014 mmol H_2_O m^−2^ s^−1^ in control conditions, while no difference was recorded for the bean from the temperate climate (*Media*). 

The induced passive heat increased the transpiration rates (Trmmol) of the maize and reported a significant difference in comparison to the control environments. The value was 4.11 ± 0.44 mmol H_2_O m^−2^ s^−1^ in OTC and 3.27 ± 0.33 mmol H_2_O m^−2^ s^−1^ in the control environments. For the factor genotypes, again, the maize from a hot and humid climate (*Huasteca*) registered the highest Trmmol, which was statistically superior to the maize from *Altiplano* and *Media* (warm–dry and temperate climates, respectively), which reported 3.20 ± 0.17 and 3.56 ± 0.16 mmol H_2_O m^−2^ s^−1^, respectively. 

The bean reported maximum values also for the plants from *Huasteca* (hot and humid climate), where the means were 10.30 ± 0.37 mmol H_2_O m^−2^ s^−1^ in control and 9.28 ± 0.5 mmol H_2_O m^−2^ s^−1^ in OTC but no statistical difference was recorded between them. For squash plants, no differences were recorded under the two factors (environment and genotypes). That means the induced passive heat did not affect them and either the genotypes. 

The maize’s intrinsic water-use efficiency (iWUE) was dramatically reduced by the induced passive heat. In comparison to the control environment, an iWUE reduction of up to 54.42% was reported. On the other hand, no difference was recorded in the maize climate. They responded as equals, no matter the characteristics of their climates, for the gas exchange parameter iWUE. 

For the bean, different responses were reported where the induced passive heat decreased the iWUE of the bean from temperate and warm–dry climates (*Media* and *Altiplano*, respectively). The first one registered the maximum value under control conditions and reduced up to 59.69 µmol CO_2_ mol^−1^ H_2_O in OTC, while the bean from *Altiplano* (warm–dry climate) reduced up to 77.33 µmol CO_2_ mol^−1^ H_2_O in comparison to the control conditions. Additionally, no difference was recorded for the bean from the hot and humid climate (*Huasteca*). That means they were not influenced by the induced passive heat. 

The induced passive heat favored the iWUE of the squash from a hot and humid climate (*Huasteca*), where a maximum value was reported in the OTC conditions with 169.16 ± 15.73 µmol CO_2_ mol^−1^ H_2_O, which was statistically superior to that reported in the control environment. Finally, no statistical differences were found in the E × G for squash from *Altiplano* and *Media* (warm–dry and temperate climates, respectively). However, the values reported in the control environment were higher than those registered in OTC conditions (Table 5).

#### 3.4.3. Effect of Induced Passive Heat on Chlorophyll Fluorescence Parameters Measured at 75 Days after Emergence of Each Crop of the Milpa 

Significant effects of the genotypes and the environment on some of the chlorophyll fluorescence parameters at 75 days after emergence using analysis of variance were observed. For the E × G interactions, all of the evaluated parameters were significant for bean, but only the maximum efficiency of the Photosystem II (Fv/Fm), the quantum yield of the Photosystem II (PhiPS2), and photochemical quenching (qP) were significant for squash. Finally, none of the parameters were significant for maize (Table S7). When the interaction was not significant, the simple effect of the environmental and genotypic factors was considered.

Table 6 shows the mentioned chlorophyll parameters for the *milpa* system at 75 days after emergence, which corresponds to the reproductive stage of the crops. 

The induced passive heat decreased the electron transport rate (ETR), maximum efficiency of the Photosystem II (Fv/Fm), the quantum yield of the Photosystem II (PhiPS2), non-photochemical quenching (qN), and alternative non-photochemical quenching (NPQ) of the maize. The results showed a significant difference in the ETR in the OTC plots, with a mean of 47.8 ± 0.06 μmol m^−2^ s^−1^, while in the control conditions, the value was 61.42 ± 0.04 μmol m^−2^ s^−1^. The induced passive heat reduced the maize ETR by up to 22.17%. At the reproductive stage, the genotypes from *Altiplano* (warm–dry climate) recorded the maximum value of ETR with 61.6 ± 2.87 μmol m^−2^ s^−1^, which was statistically higher than the reported for the maize from temperate and hot and humid climates (*Media* and *Huasteca*, respectively), where no differences were observed. 

The induced passive heat greatly improved the ETR of the bean from a hot and humid region (*Huasteca*), with the greatest value reported in the OTC environments at 61.69 ± 0.45 μmol m^−2^ s^−1^, which was statistically greater than the control. No differences were recorded for the bean from the warm–dry and temperate climates (*Altiplano* and *Media*, respectively) in comparison to the two environments. However, the bean from the warm–dry climate (*Altiplano*) reported the lowest values of ETR in OTC. The bean responded differently in dependence on their climate. 

The passive heat also reduced the ETR of the squash with a significant difference. A reduction of 38.76% of the squash ETR was reported by the effect of the heat conditions. For the factor genotypes, the squash from temperate (*Media*) reported the maximum mean with 94.6 ± 10.29 μmol m^−2^ s^−1^, which was statistically superior to the results registered in *Huasteca* and *Altiplano*. The last one reported the lowest ETR with 34.76 ± 6.24 μmol m^−2^ s^−1^. That means the squash responded differently depending on the climate. 

The maize reported the maximum mean of Fv/Fm under control conditions with 0.87 ± 0.001, which was statistically higher than that reported in OTC. The climate features of the maize, on the other hand, showed no variations because they both responded equally well to the passive heat. 

Different responses were reported in the E × G for the bean, where the induced passive heat affected the plants from the hot and humid climate (*Huasteca*) more. They decreased the Fv/Fm of the control environment (0.8 ± 0.002), which is the maximum value in comparison to OTC. For the bean from *Altiplano* (warm–dry climate) and *Media* (temperate), no differences were recorded under the two conditions. However, higher values of Fv/Fm were reported under the control conditions. 

The squash plants showed different responses in the E × G where the induced passive heat increased the Fv/Fm at the reproductive stage for the plants from hot and humid climates (*Huasteca*). They significantly increased the Fv/Fm with a maximum of 0.75 ± 0.005 in OTC vs. 0.66 ± 0.02. The squash from *Huasteca* was the only one that benefitted from the effect of the induced passive heat, while the others from *Altiplano* and *Media* (warm–dry and temperate climates, respectively) responded equally, with no differences in the two environments. 

Compared to the control environments, the maize lost up to 41.66% of its PhiPS2 when exposed to passive heat. The climate features of the maize for the variable PhiPS2 showed no variations because they both responded equally well to the passive heat. 

The bean responded differently to the effect of the induced passive heat for PhiPS2. The bean from *Media* (temperate) benefited up to 78.94% of PhiPS2 in OTC in comparison to control environments. They were statistically higher than those reported in the control environment. Bean from *Altiplano* and *Huasteca* (warm–dry and hot and humid climates, respectively) showed no differences in PhiPS2. However, maximum values were reported in the control environments. 

Additionally, the squash from the temperate and hot and humid climates (*Media* and *Huasteca*, respectively) significantly increased its PhiPS2. They reported 0.49 ± 0.05 in OTC vs. 0.26 ± 0.04 in control (*Media*) and 0.37 ± 0.02 in OTC vs. 0.16 ± 0.008 in control (*Huasteca*). In addition, no difference was recorded for the squash from warm–dry climates (*Altiplano*). The non-photochemical quenching (qN) and alternative non-photochemical quenching (NPQ) of the maize were reduced by up to 7.86% and 15.53%, respectively, by the induced passive heat. However, no differences were recorded for the factor genotypes of the maize for the two non-photochemical parameters.

At the reproductive stage, the induced passive heat increased the qN of the bean from the hot and humid climate (*Huasteca*). They registered the maximum in OTC conditions with 0.86 ± 0.006, which was statistically higher than the reported in control with 0.63 ± 0.04. On the other hand, no differences were recorded for beans from *Altiplano* and *Media* (warm–dry and temperate climates, respectively), but the values reported in the control were higher than those recorded in OTC plots. In addition, for the alternative non-photochemical quenching (NPQ), the beans responded differently and were affected by the induced passive heat. The bean from the temperate climate (*Media*) significantly reduced their NPQ to 0.264 in comparison to control environments. No differences were reported for the bean from *Altiplano* and *Huasteca* (warm–dry and hot and humid climates, respectively). 

The squash’s qN dropped with passive heat, with a difference of 0.16 compared to the control, whereas there were no variations between the two conditions for NPQ. For the factor genotypes, the squash from *Media* (temperate) and *Huasteca* (hot and humid) recorded the maximum values of qN and NPQ with 0.62 ± 0.06 and 0.77 ± 0.02, respectively, 1.09 ± 0.13 and 1.53 ± 0.11, respectively. The lowest values were reported for squash from *Altiplano* (warm–dry) for the two non-photochemical parameters.

At 75 days after emergence, the photochemical quenching (qP) parameter was the only one where the E × G was significant for maize. In this approach, the induced passive heat significantly decreased the qP of the maize. The maximum means were reported under the control environment for the three genotypes with 0.44 ± 0.008; 0.44 ± 0.008, and 0.45 ± 0.008 for *Altiplano* (warm–dry), *Media* (temperate), and *Huasteca* (hot and humid), respectively, while in OTC they were 0.32 ± 0.002, 0.25 ± 0.008, and 0.34 ± 0.001. That means the induced passive heat affected the qP parameters, and the maize responded differently to the characteristics of their climates (Table 6).

#### 3.4.4. Effect of Induced Passive Heat on Gas Exchange Parameters Measured at 75 Days after Emergence of Each Crop of the Milpa 

Significant effects of the genotypes and the environment on the gas exchange parameters 75 days after the emergence of the crops using analysis of variance were observed. For maize, only the E × G of the transpiration rates (Trmmol) was significant (Table S8). When the interaction was not significant, the simple effect of the environmental and genotypic factors was considered.

Table 7 shows the four gas exchange parameters used to evaluate the effect of the induced passive heat on the *milpa* system from different climates 75 days after emergence. The passive induced heat did not affect the photosynthetic rate (photo) of the maize at the reproductive stage because no difference was recorded between the two environments. However, the maize from *Huasteca* (hot and humid) and *Media* (temperate) reported the maximum photosynthetic rate with 61.29 ± 2.33 µmol CO_2_ m^−2^ s^−1^ and 57.69 ± 2.39 µmol CO_2_ m^−2^ s^−1^, respectively, which was statistically different to the reported from *Altiplano* (warm–dry). The last ones registered the lowest photosynthetic rate with 40.44 ± 3.23 µmol CO_2_ m^−2^ s^−1^. 

The bean and squash were affected by the induced passive and significantly reduced their CO_2_ assimilation rate. The genotypes responded differently to the effect of passive heat, where the squash and bean from hot and humid climates (*Huasteca*) reported the maximum values, with 19.89 ± 1.76 µmol CO_2_ m^−2^ s^−1^ and 12.75 ± 1.86 µmol CO_2_ m^−2^ s^−1^, respectively, while those from *Altiplano* (warm–dry) recorded the lowest with 6.14 ± 1.24 µmol CO_2_ m^−2^ s^−1^ for squash and 4.58 ± 0.56 for the beans. That means the beans and squash from warm–dry climates were the most affected in the stage of reproduction for CO_2_ assimilation. 

For maize and bean, there was no variation in stomatal conductance (Cond) between genotypes and environments. That suggests the maize and bean reacted in the same way to the passive heat. In contrast, the squash benefited from their stomatal conductance under the effect of the passive heat. 

An increase of 0.21 mmol H_2_O m^−2^ s^−1^ in OTC was recorded for the squash. The E × G was significant for transpiration rates (Trmmol) of the maize; however, no differences in statistics were observed. In addition, the maize from warm–dry climate (*Altiplano*) was the most affected and reported the lowest value in OTC with 3.11 ± 0.69 mmol H_2_O m^−2^ s^−1^ and 4.46 ± 0.31 mmol H_2_O m^−2^ s^−1^ in control conditions. 

The bean from hot and humid climates (*Huasteca*) showed the minimum value of transpiration rates (Trmmol), with 3.68 ± 0.28 mmol H_2_O m^−2^ s^−1^, while those from temperate climates (*Media*) reported the highest values, with 4.71 ± 0.35 mmol H_2_O m^−2^ s^−1^. On the other side, the induced passive heat significantly impacted the Trmmol of the beans. A reduction of 1 mmol H_2_O m^−2^ s^−1^ was reported comparing the two conditions. In contrast, the induced passive heat significantly increased the Trmmol of the squash. They reported an increase of 1.49 mmol H_2_O m^−2^ s^−1^.

In both environments, the maize intrinsic water-use efficiency (iWUE) was affected by the induced passive heat with a significant difference. They decreased by up to 26.48% the iWUE in comparison to the control plots. Additionally, the crops bean and squash showed a reduction in their iWUE with significant differences. They reported a reduction of up to 43.56% and 57.43% for bean and squash, respectively. Furthermore, the genotypes of bean and squash were affected by the passive heat effect and behaved differently. The highest iWUE was reported for the squash from hot and humid climate (*Huasteca*) with 44.89 ± 5.15 µmol CO_2_ mol^−1^ H_2_O, which was statistically superior to those reported from *Media* and *Altiplano* (temperate and warm–dry climates, respectively). The last reported the lowest iWUE, with 11.52 ± 2.34 µmol CO_2_ mol^−1^ H_2_O. Additionally, the beans from hot and humid climates (*Huasteca*) reported the maximum value of iWUE, with 47.56 ± 7.16 µmol CO_2_ mol^−1^ H_2_O, while no differences were reported for those from temperate and warm–dry climates (*Media* and *Altiplano*, respectively) (Table 7).

### 3.5. Correlation among Abiotic Variables and Morphological, and Yield Parameters of the Milpa 

As morphological parameters are very interdependent for the *milpa* system, the correlations between them and the abiotic variables that are necessary for determining the overall performances of the crops have been established. An increase in temperature and accumulated heat units leads to an increased plant height of maize and bean but a decreased squash plant height. Additionally, the increase in the abiotic variables leads to an increase in the rate of growth of maize but a decrease in the RG of the bean and squash. A significant but negative correlation was reported for the number of leaves of the squash, where the increase of the abiotic variables decreased the number of leaves considerably (Figure 6A). In addition, an increase in temperature was negatively correlated with the yield of the *milpa*. This negative correlation was significant for the squash, confirming that they were the most affected by the induced passive heat, meaning that the induced passive heat will considerably decrease the yield of the *milpa* system (Figure 6B).

### 3.6. Correlation among Abiotic Variables and Various Photosynthetic Parameters of the Milpa System 

Significant and negative correlations were reported for the maximum efficiency of the Photosystem II (Fv/Fm) for beans, intrinsic water-use efficiency (iWUE), and non-photochemical quenching (qN) for maize, with the data of photosynthesis, recorded 45 days after emergence in each crop, while a positive and significant correlation was only obtained for the stomatal conductance (Cond) of the maize. The possible reason may be the increase in stomatal opening that is very directly associated with the photosynthetic rate. An increase in the abiotic variables leads to a reduction in the photosynthetic capacity of the *milpa* system (Figure S1A).

At the reproductive stage of the crops, more chlorophyll fluorescence parameters correlate significantly and negatively with the abiotic variables and only for maize. Those parameters were the efficiency of the Photosystem II (Fv/Fm), photochemical quenching (qP), and non-photochemical quenching (qN). The possible reason may be related to the physiology of the maize, which is the one growing vertically and directly in contact with the light sun. In contrast, positive and significant correlations were shown in squash plants with some gas exchange parameters (stomatal conductance and transpiration rates). That means the increase in the abiotic variables promoted the gas exchange of the squash. The possible reason may also be related to the management of the *milpa* system, where the maize plants protect the beans and squash plants against direct contact with the sun’s light because even though the correlations of the bean gas exchange parameters were not significant, they were positive (Figure S1B).

## 4. Discussion

In this study, we investigated the influence of increased air temperature (abiotic variable) under climate change scenarios on *milpa* systems from distinct climates in the state of SLP (Mexico). For the experiment, OTC was used to simulate the induced passive heat and allow evaluation of the *milpa* systems and their responses under a prognostic increase in temperature. In this approach, the employment of OTC appears to have resulted in accurate temperature projections [25]. Our warming methods resulted in a maximum increase of 1.8 °C in the mean daily air temperature for OTC at 15 cm above the soil, while an increase of 3.65 °C was reported at 150 cm above the soil. This was within the expected 1–3 °C increase in global warming by the late twenty-first century [36,37,38]. Moreover, because there were more Growing Day Degrees (GDD) found in OTC than in control (Figure 3A,B), most of the morphological parameters benefited the crops of the *milpa* system [39]. In addition, the *milpa* system from a hot and humid climate (*Huasteca*) responded with more plant height, rate of growth, width of leaf, height to ear insertion, number of leaves per plant, leaf area, leaf length, and number of flowers per plant. The possible reason may be related to the adaptation characteristics of the genotypes to their climate, where more mean temperature and precipitation are reported (Figure 1). Then this study, therefore, indicates that genotypes from different regions responded differently to the temperature effect [20]. In our case, the three different *milpa* systems responded differently to the effect of induced passive heat and increased most of the morphological parameters of the crops that conformed to their *milpa* systems. Furthermore, the induced passive heat accelerated the time to flowering for the maize. It is important to remember that those variables were only measured for maize crops. Our results are consistent with those of [40,41,42], who stated that high temperatures could accelerate floret differentiation, reduce pollen shedding duration, and delay silking. The maize from a hot and humid climate (*Huasteca*) took more time to complete their reproductive stage. In this case, those from warm–dry (*Altiplano*) and temperate (*Media*) climates completed their reproductive stages faster than those from the hot and humid climates. The results can be associated with the reason that the materials of dry and temperate (*Altiplano* and *Media*) environments with strong variation in the date of sowing have greater phenotypic plasticity than those of relatively more stable environments such as *Huasteca* (hot and humid). Furthermore, the current study clearly demonstrated that the induced passive heat during the intercropping *milpa* system resulted in yield loss. As a result, the squash was the most severely affected (Figure 4), with a loss of up to 91.94% of its yield recorded. The reason was that they used to abort under the OTC plots, and that reduced their yield considerably. Most of the cucurbits are perishable and very sensitive to unpredictable climatic changes. Environmental stress, such as increasing (high) temperature, is thought to be one of the major limiting factors in enhancing Cucurbitaceous vegetable productivity [43]. Additionally, the maize reported a reduction of up to 43.31% of the yield parameter. As mentioned in our OTC conditions, heat stress is a multifaceted challenge of strength (temperature degrees), duration, and rate of temperature augmentation and affects the *milpa* system. The reduction of the yield parameters is well correlated with the increase of the abiotic variables (Figure 6B). Our results are in concordance with [3,10,44,45], who stated that under climate change, the temperature is expected to increase, and maize production could be heavily and negatively impacted by climate change [46]. The negative impact of the related effects of climate change on maize in Mexico has been largely studied [20,47,48,49,50]. Abiotic stresses have also been related to the effects of climate change in Mexico and will negatively affect maize germination, seedlings, growth and reproduction, and yield [49,51]. The bean was the least affected by the induced passive heat because only those from hot and humid climates (*Huasteca*) reduced their yield. This result can be associated with the fact that the experiment was carried out in an area with a vapor pressure deficit greater than that which it normally faces in its region of origin. In the same way, for the ones from *Media* (temperate) and *Altiplano* (warm–dry), no differences were registered between control and OTC.

With the objective of detecting how the yields of the three crops were affected by the induced passive heat, we made a correlation between the abiotic variables and the values of the obtained yields for each crop. We discovered that increasing the abiotic variables significantly reduced the squash, as well as the maize and bean yields. We found that squash is the most affected by the warming effect (Figure 2). During the experiment, the squash plants used to abort their flowers due to the consequences of the warming effect. That explains the loss of the yield for the squash plants, and it is reported that high temperature is thought to be one of the major limiting factors in enhancing Cucurbitaceous vegetable productivity [43].

Chlorophyll fluorescence analysis has become one of the most potent and extensively used tools in plant physiology research. The chlorophyll fluorescence parameters were represented by the measurement of photosystem II, which is found in the thylakoid membranes and is intimately linked to instant plant damage caused by stress conditions [52]. In our study, the induced passive heat affected the chlorophyll fluorescence of the *milpa* system differently at the early and late stages of the crops. The induced passive heat increased the ETR, Fv/Fm and decreased the PhiPS2, qN, NPQ, and qP of the maize plants. Furthermore, the response was different for bean and squash. The ETR increased by the effect of the induced passive heat in beans and squash, while Fv/Fm, PhiPS2, and qN reduced their values under the effect of the passive heat for the two crops. On the other hand, the qP was increased by the passive heat effect. Taking into account that under stress conditions, Fv/Fm, PhiPS2, and qP are the most important parameters [53], our results are in accordance with that because the passive heat decreased those parameters for maize, bean, and squash (except qP). Our results agree with [54] findings, that the qP of the two maize varieties decreased significantly under warming treatment. [55] indicated some differences in the measurement of the physiological parameters of bean (*Phaseolus vulgaris* L.) due to the chambers effects, which are certainly caused by the physical structure of the OTC. In our investigation, the photochemical quenching was affected by the passive induced heat. According to [24], OTC can reduce up to 25% of the photosynthetically active radiation and increase the air temperature. These results are consistent with ours, as there was a reduction of photochemical quenching and an increase in air temperature with respect to the control environment. [56] reported similar results to ours for maize grown in high temperatures, keeping in mind that 20/25 °C is close to our mean diurnal temperature during the experiment. Additionally, our results were similar to other researchers, such as [57], who found that an increase in temperature reduces photosynthesis in maize leaves. In the same way, [58] reported that photoinhibition occurs when light energy exceeds the amount of energy used for photosynthesis, characterized by a decline in the PhiPS2. In addition, [59] and [60] reported that the photosynthetic apparatus depends on the severity and duration of the stress.

In the current study, the gas exchange effect under passive heat stress has been reported to have different responses in the crops at 45 and 75 days after emergence. The photosynthetic rate, or CO_2_ assimilation, and intrinsic water-use efficiency were affected by the passive heat for all the crops in the system, while the stomatal conductance and transpiration rate were not affected at the early stage. Furthermore, the induced passive heat did not affect the maize photosynthetic rate at the reproductive stage, while the three other gas exchange parameters were affected. These results coincide with the analysis by [61], in which they conclude that increased temperature while maintaining soil moisture, increases rainfed agriculture suitability in semiarid temperate regions (equivalent to *Altiplano* region). In our investigation, we show that a change in the development of photosynthesis apparatus exists in the milpa system for successful adaptation as measured by reduced CO_2_ assimilation and higher water-use efficiency appears to be involved in crop adaptation success [29]. In addition, CO_2_ exchange parameters act as chief indicators of plant growth due to their direct link to net productivity [62]. The maize plants from a hot and humid climate (*Huasteca*) reported the highest values of the gas exchange parameters. That means they responded differently to the other genotypes. The genotypes from the warm–dry climate (*Altiplano*) were the most affected, even for the two other crops. Studies showed that early closure of stomata and decreasing transpiration were found to be thermal sensitive in maize plants grown at high temperatures [63], as we reported in our study. In various crop species, such as soybean, tobacco, and grape, global warming has been found to increase stomatal frequency while decreasing stomatal size, though no effect has been recorded in maize [64,65,66].

In our investigation, correlations were made between abiotic variables and photosynthetic variables at early and reproductive stages. Our results showed that the increase in the values of the abiotic variables leads to reduced specific variables such as Fv/Fm and qN for bean and maize at an early stage, while at the late stage, they were Fv/Fm, qP, and qN for maize. On the other hand, the increase in the values of the abiotic variables leads to improved stomatal conductance and transpiration rate of the squash (Figure S1A,B). That means, in our case, the mentioned parameters were the most affected by the passive heat of the evaluated crops in our *milpa*. 

This study analyzed the effect of induced passive heat, which aims to simulate a scenario of global warming due to climate change, in the *milpa* system from different climates of the state of SLP (México). In the *milpa* system, maize is the most important crop, and in Mexico, maize have an abundant genetic variability in all the country. In this approach, maize was originally categorized into different races and genotypes that have been related to particular environmental conditions. Furthermore, Mexican maize was classified by [67] based on rainfall, photoperiod, and, most importantly, the temperature of local adaptations or origins. These findings have crucial implications for thinking about the effects of climate change adaptation on maize in the country in general and the state of San Luis Potosí in particular because they highlight a way to contrast the negative effects of climate change while taking local conditions into account [20]. In our previous experiences (germination and emergence) [20], the maize genotypes from hot and humid climates were the most affected. However, in the complete experience from emergence to yield, the maize from *Huasteca* (hot and humid climate) reported the highest values in the growth and development parameters, photosynthetic, and yield. The reason could be related to their origin environments’ specific local adaptation. This is the first report to investigate the effects of warming on the *milpa* system, taking into account the variability of the climates in SLP. Smallholders, in particular, are among those most affected by climate change. Our report is also a pioneering experiment in the state. From here, more investigation could be undertaken in each region using OTC as a model to simulate the increase in temperature.

## 5. Conclusions

The OTC structures increase the abiotic variables (minimum, maximum, mean daily temperature, and accumulated heat units). The morphological parameters of the crops *milpa* system increased under the warming effect. When exposed to an increase in temperature, the milpa system responded differently depending on its origins, which is related to the effect of climate change. Furthermore, the *milpa* from a hot and humid climate (*Huasteca*) was the least affected by the induced passive heat. In contrast, the warming considerably delayed the yield parameters of the *milpa* crops. The yield parameters had a significant impact on the squash, whereas the bean benefited the most. For each crop, the warming had a varied impact on gas exchange and chlorophyll fluorescence. However, at an early stage (45 days after emergence), maximum efficiency of the photosystem II (Fv/Fm) and non-photochemical quenching (qN) for bean and maize were decreased, while at the reproductive stage (75 days after emergence), they were Fv/Fm, qN, and photochemical quenching (qP) for maize. The stomatal conductance and transpiration rate of the squash were enhanced by the warming effect.

## Figures and Tables

**Table 1:** Effect of induced passive heating on plant height, rate of growth, width leaf and stem thickness of maize; number of leaves per plant, plant height, stem thickness and rate of growth of squash and stem thickness and number of leaves per plant on bean from different climate of the state of San Luis Potosí (Mexico).

Plant Height (m)					
Crops		Genotype			
	Environment	*Altiplano*	*Media*	*Huasteca*	Significance
Maize	OTC	2.46 ± 0.06b	2.47 ± 0.05b	2.79 ± 0.05a	E × G ** E * G ***
	Control	1.94 ± 0.07c	1.79 ± 0.04c	2.46 ± 0.03b	
	LSD	0.24			
	Rate of growth (m.day^−1^)				
		Genotype			E × G *** E ** G ***
	Environment	*Altiplano*	*Media*	*Huasteca*	
	OTC	0.01 ± 0.0005b	0.01 ± 0.0004b	0.01 ± 0.0004a	
	Control	0.008 ± 0.0005c	0.008 ± 0.0005c	0.01 ± 0.0003ab	
	LSD	0.009			
	Width leaf (cm)				
		Genotype			E × G ** E ** G ***
	Environment	*Altiplano*	*Media*	*Huasteca*	
	OTC	9.73 ± 0.24b	7.97 ± 0.19c	9.46 ± 0.18b	
	Control	11.08 ± 0.19a	11.10 ± 0.11a	11.32 ± 0.26a	
	LSD	0.86			
	Stem Thickness (mm)				
		Genotype			E × G ** E ** G ***
	Environment	*Altiplano*	*Media*	*Huasteca*	
	OTC	33.86 ± 0.9ab	30.05 ± 0.58c	33.64 ± 0.77ab	
	Control	34.85 ± 0.65a	32.3 ± 0.95abc	30.98 ± 0.63bc	
	LSD	3.22			
Bean	Number of leaves per plant				
		Genotype			E × G ** E * G ***
	Environment	*Altiplano*	*Media*	*Huasteca*	
	OTC	21.1 ± 0.52d	25.6 ± 4.39cd	34.5 ± 0.55bc	
	Control	37 ± 1.69b	29 ± 1.88bcd	56.6 ± 3.64a	
	LSD	11.3			
	Stem thickness (mm)				
		Genotype			E × G ** E ** G *
	Environment	*Altiplano*	*Media*	*Huasteca*	
	OTC	5.56 ± 0.25b	6.32 ± 0.16b	10.8 ± 0.51a	
	Control	6.32 ± 0.35b	6.74 ± 0.22b	9.45 ± 0.54a	
	LSD	1.62			
Squash	Number of leaves per plant				
			Genotype		E × G ** E ** G **
	Environment	*Altiplano*	*Media*	*Huasteca*	
	OTC	43.33 ± 0.87b	24.5 ± 4.45c	38.33 ± 2.56b	
	Control	60.17 ± 1.95a	57.67 ± 1.22a	63.67 ± 0.87a	
	LSD	11.09			
	Plant height (cm)				
		Genotype			E × G *** E * G **
	Environment	*Altiplano*	*Media*	*Huasteca*	
	OTC	99.07 ± 0.59b	98 ± 2.11b	96.7 ± 0.56b	
	Control	112.93 ± 3.42a	97.7 ± 2.24b	109.53 ± 2.69a	
	LSD	10.37			
	Stem thickness (mm)				
			Genotype		E × G *** E * G ***
	Environment	*Altiplano*	*Media*	*Huasteca*	
	OTC	18.96 ± 0.27b	23.75 ± 2.67ab	17.09 ± 2.65b	
	Control	23.8 ± 0.13ab	24.36 ± 0.34ab	27.97 ± 0.78a	
	LSD	7.45			
	Rate of growth (cm.day^−1^)				
		Genotype			E × G ** E * G **
	Environment	*Altiplano*	*Media*	*Huasteca*	
	OTC	0.6 ± 0.07b	0.6 ± 0.01b	0.6 ± 0.01b	
	Control	0.63 ± 0.03b	0.62 ± 0.01b	0.85 ± 0.03a	
	LSD	0.087			

OTC: Open-top Chamber. LSD: Least Significant Difference; E: Environment; G: Genotype; * *t*-test, *p* < 0.05; ** *t*-test, *p* < 0.01, and *** *t*-test, *p* < 0.001; The letters a, b, c, and d indicate significant differences according to the Tukey test (*p* < 0.05); (*n* = 20 for maize, *n* = 10 for bean and *n* = 6 for squash). The values are the means ± SE (standard error).

**Table 2:** Effect of the induced passive heating on morphological variables for the *milpa* system from different climates [*Altiplano* (warm–dry), *Media* (temperate) and *Huasteca* (hot and humid climate)] of the state of San Luis Potosí (Mexico).

Crops	Leaf Number Per Plant				
Maize	Genotype				
		*Altiplano*	*Media*	*Huasteca*	Significance
	OTC	12 ± 0.25b			E × G(ns) E ** G *
	Control	13 ± 0.26a			
	LSD	0.52			
		12 ± 0.3b	11 ± 0.2b	14 ± 0.2a	
	LSD	0.77			
	Leaf length (cm)				
		Genotype			
		*Altiplano*	*Media*	*Huasteca*	
	OTC	96.7 ± 1.37a			E × G(ns) E(ns) G **
	Control	95.1 ± 1.26a			
	LSD	3.1			
		93 ± 1.22b	90.4 ± 1.42b	103.9 ± 1.35a	
	LSD	4.5			
	Leaf area (cm^2^)				
		Genotype			
		*Altiplano*	*Media*	*Huasteca*	
	OTC	664.3 ± 14.62b			E × G(ns) E ** G ***
	Control	796.97 ± 14.12a			
	LSD	33.5			
		723.12 ± 16.9b	658.54 ± 15.6c	810.29 ± 20.9a	
	LSD	49.3			
	Days for female flowering per plot				
		Genotype			
		*Altiplano*	*Media*	*Huasteca*	
	OTC	61.7 ± 2.93a			E × G(ns) E(ns) G **
	Control	57.2 ± 2.27a			
	LSD	6.2			
		55.7 ± 2.01b	55.2 ± 2.8b	67.2 ± 1.51a	
	LSD	9.6			
	Days for male flowering per plot				
		Genotype			
		*Altiplano*	*Media*	*Huasteca*	
	OTC	57.5 ± 2.67b			E × G(ns) E * G **
	Control	63.7 ± 1.3a			
	LSD	4.4			
		56.5 ± 2.01b	59 ± 2.81b	66.2 ± 1.51a	
	LSD	6.7			
	Height to ear insertion (m)				
		Genotype			
		*Altiplano*	*Media*	*Huasteca*	
	OTC	1.41 ± 0.03a			E × G(ns) E * G **
	Control	0.98 ± 0.03b			
	LSD	0.03			
		1.23 ± 0.05ab	1.11 ± 0.05b	1.25 ± 0.04a	
	LSD	0.05			
Bean	Number of flowers per plant				
		Genotype			
		*Altiplano*	*Media*	*Huasteca*	
	OTC	12.9 ± 0.76b			E × G(ns) E * G **
	Control	20.2 ± 0.43a			
	LSD	1.67			
		14 ± 1.49b	19.6 ± 0.66a	18.08 ± 0.72ab	
	LSD	2.46			
	Plant height (cm)				
		Genotype			
		*Altiplano*	*Media*	*Huasteca*	
	OTC	37.96 ± 0.8a			E × G(ns) E (ns) G *
	Control	36.68 ± 0.71a			
	LSD	2.42			
		35.35 ± 1.05b	37.32 ± 0.71ab	39.28 ± 0.77a	
	LSD	3.56			
	Rate of growth (cm day^−1^)				
		Genotype			
		*Altiplano*	*Media*	*Huasteca*	
	OTC	0.11 ± 0.007b			E × G(ns) E * G **
	Control	0.16 ± 0.007a			
	LSD	0.02			
		0.13 ± 0.01ab	0.12 ± 0.01b	0.16 ± 0.01a	
	LSD	0.03			
Squash	Number of flowers per plant				
		Genotype			
		*Altiplano*	*Media*	*Huasteca*	
	OTC	5.11 ± 0.75b			E × G(ns) E ** G *
	Control	13.44 ± 1.15a			
	LSD	4.3			
		12.58 ± 1.94a	8.08 ± 1.3a	7.17 ± 1.32a	
	LSD	6.35			

OTC: Open-top chamber; LSD: Least Significant Difference; E: Environment; G: Genotype; ns: no significant; * *t*-test, *p* < 0.05; ** *t*-test, *p* < 0.01, and *** *t*-test, *p* < 0.001; The letters a, b, c indicate significant differences according to the Tukey test (*p* < 0.05). The values are the means ± SE (standard error).

**Table 3:** Effect of the induced passive heating on yield and its component variables of the maize from different climates [*Altiplano* (warm–dry), *Media* (temperate) and *Huasteca* (hot and humid climate)] of the state of San Luis Potosí (Mexico).

Crop	Number of Cobs per Plant				
Maize		Genotype			
		*Altiplano*	*Media*	*Huasteca*	Significance
	OTC	1.26 ± 0.06b			E × G (ns) E ** G *
	Control	2.2 ± 0.08a			
	LSD	0.07			
		1.55 ± 0.1b	1.75 ± 0.1ab	1.9 ± 0.13a	
	LSD	0.1			
	Cob length (cm)				
		Genotype			
		*Altiplano*	*Media*	*Huasteca*	
	OTC	14.36 ± 0.29b			E × G (ns) E ** G *
	Control	18.31 ± 0.14a			
	LSD	0.65			
		16.08 ± 0.43a	16.49 ± 0.38a	16.42 ± 0.45a	
	LSD	0.95			
	Number of grains per row				
		Genotype			
		*Altiplano*	*Media*	*Huasteca*	
	OTC	30.65 ± 1b			E × G (ns) E * G *
	Control	37.6 ± 0.5a			
	LSD	2.21			
		32.22 ± 0.75b	33.42 ± 0.96b	36.72 ± 1.46a	
	LSD	3.24			
	100 grains weight per plot (g)				
		Genotype			
		*Altiplano*	*Media*	*Huasteca*	
	OTC	42.21 ± 2.76b			E × G (ns) E ** G *
	Control	48.98 ± 1.71a			
	LSD	4.22			
		48.39 ± 2.14a	49.55 ± 0.43a	38.84 ± 2.71b	
	LSD	6.48			
	Yield (t ha^−1^)				
		Genotype			
		*Altiplano*	*Media*	*Huasteca*	
	OTC	3.05 ± 0.42b			E × G (ns) E ** G *
	Control	5.38 ± 0.53a			
	LSD	4.22			
		5.08 ± 0.73a	4.62 ± 0.48ab	2.93 ± 0.61b	
	LSD	1.95			

OTC: Open-top chamber; LSD: Least Significant Difference; E: Environment; G: Genotype; ns: no significant; * *t*-test, *p* < 0.05; ** *t*-test, *p* < 0.01; The letters a, b indicate significant differences according to the Tukey test (*p* < 0.05). The values are the means ± SE (standard error).

**Table 4:** Chlorophyll fluorescence parameters measured in different system of milpa from different environment under induced passive heat and controlled ambient at 45 days after emergence of each crop.

		Electron Transport Rate (ETR) (μmol m^−2^ s^−1^)			
		Genotype			Significance
Crops	Environment	*Altiplano*	*Media*	*Huasteca*	
Maize	OTC	58.2 ± 3.9a			E × G(ns) E(ns) G **
	Control	61.1 ± 3.3a			
	LSD	0.06			
		54.2 ± 3.3b	53.1 ± 3.5b	71.7 ± 5.2a	
	LSD	0.08			
Bean	OTC	15.2 ± 1.7d	23.5 ± 2.58c	51.4 ± 0.5a	E × G ** E * G ***
	Control	12.3 ± 0.5d	24.4 ± 1.11c	41.4 ± 1.01b	
	LSD	0.24			
Squash	OTC	40.93 ± 6.65b	42.67 ± 10.39b	20.39 ± 1.6c	E × G *** E ** G ***
	Control	18.26 ± 1.51c	81.03 ± 1.29a	62.13 ± 1.14ab	
	LSD	24.34			
	Maximum efficiency of the Photosystem II (Fv/Fm)				
Maize		Genotype			
		*Altiplano*	*Media*	*Huasteca*	E × G(ns) E ** G(ns)
	OTC	0.75 ± 0.008a			
	Control	0.72 ± 0.005b			
	LSD	0.02			
		0.75 ± 0.01a	0.73 ± 0.007a	0.73 ± 0.006a	
	LSD	0.03			
Bean	OTC	0.59 ± 0.01b			E × G(ns) E ** G(ns)
	Control	0.66 ± 0.02a			
	LSD	0.04			
		0.62 ± 0.02a	0.63 ± 0.01a	0.63 ± 0.02a	
	LSD	0.06			
Squash	OTC	0.51 ± 0.01b			
	Control	0.56 ± 0.02a			
	LSD	0.04			
		0.51 ± 0.01a	0.55 ± 0.02a	0.55 ± 0.02a	
	LSD	0.06			
Maize		Quantum yield of the Photosystem II (PhiPS2)			
		Genotype			
		*Altiplano*	*Media*	*Huasteca*	E × G(ns) E *** G **
	OTC	0.08 ± 0.006a			
	Control	0.05 ± 0.005b			
	LSD	0.02			
		0.06 ± 0.008b	0.06 ± 0.005b	0.08 ± 0.008a	
	LSD	0.03			
Bean	OTC	0.15 ± 0.01b	0.05 ± 0.001d	0.11 ± 0.001b	E × G** E *** G ***
	Control	0.26 ± 0.01a	0.07 ± 0.001c	0.16 ± 0.01b	
	LSD	0.04			
Squash	OTC	0.34 ± 0.08ab	0.35 ± 0.03ab	0.31 ± 0.04ab	E × G * E(ns) G(ns)
	Control	0.52 ± 0.03a	0.3 ± 0.04ab	0.27 ± 0.01b	
	LSD	0.19			
		Non-photochemical quenching (qN)			
Maize		Genotype			
		*Altiplano*	*Media*	*Huasteca*	E × G(ns) E *** G *
	OTC	0.84 ± 0.01b			
	Control	0.91 ± 0.003a			
	LSD	0.03			
		0.89 ± 0.006a	0.89 ± 0.01a	0.85 ± 0.02a	
	LSD	0.04			
Bean	OTC	0.25 ± 0.005c	0.23 ± 0.008c	0.45 ± 0.002a	E × G *** E *** G ***
	Control	0.33 ± 0.009b	0.46 ± 0.012a	0.49 ± 0.009a	
	LSD	0.04			
Squash	OTC	0.33 ± 0.07bc	0.22 ± 0.04cd	0.47 ± 0.01ab	E × G *** E(ns) G ***
	Control	0.16 ± 0.04d	0.58 ± 0.02a	0.57 ± 0.02a	
	LSD	0.18			
Maize		Photochemical quenching (qP)			
		Genotype			
		*Altiplano*	*Media*	*Huasteca*	
	OTC	0.32 ± 0.02a			E × G(ns) E(ns) G *
	Control	0.33 ± 0.01a			
	LSD	0.04			
		0.3 ± 0.02b	0.3 ± 0.01b	0.37 ± 0.02a	
	LSD	0.05			
Bean	OTC	0.42 ± 0.07ab	0.55 ± 0.01a	0.32 ± 0.02ab	E × G ** E(ns) G(ns)
	Control	0.42 ± 0.06ab	0.31 ± 0.06b	0.54 ± 0.01a	
	LSD	0.18			
Squash	OTC	0.5 ± 0.09ab	0.68 ± 0.03a	0.56 ± 0.03a	E × G ** E * G *
	Control	0.66 ± 0.06a	0.42 ± 0.1ab	0.24 ± 0.01b	
	LSD	0.22			
Maize		Alternative non-photochemical quenching (NPQ)			
		Genotype			
		*Altiplano*	*Media*	*Huasteca*	E × G(ns) E *** G **
	OTC	1.55 ± 0.06b			
	Control	1.84 ± 0.04a			
	LSD	0.06			
		1.8 ± 0.04a	1.76 ± 0.05a	1.53 ± 0.08b	
	LSD	0.08			
Bean	OTC	0.51 ± 0.02c	0.95 ± 0.001b	1.36 ± 0.01a	E × G *** E(ns) G ***
	Control	1.03 ± 0.001b	1.03 ± 0.001b	0.89 ± 0.07b	
	LSD	0.16			
Squash	OTC	0.73 ± 0.11a			E × G(ns) E(ns) G ***
	Control	0.89 ± 0.1a			
	LSD	0.16			
		0.42 ± 0.09b	0.89 ± 0.12a	1.13 ± 0.02a	
	LSD	0.23			

OTC: Open-top chamber; LSD: Least Significant Difference; E: Environment; G: Genotype; ns: no significant; * *t*-test, *p* < 0.05; ** *t*-test, *p* < 0.01, and *** *t*-test, *p* < 0.001; The letters a, b, c, and d indicate significant differences according to the Tukey test (*p* < 0.05). The values are the means ± SE (standard error).

**Table 5:** Effect of induced passive heating on gas exchange parameters of the *milpa* system at 45 days after emergence from different climate of the state of San Luis Potosí (Mexico).

	Photosynthetic Rate (µmol CO_2_ m^−2^ s^−1^)				
Crops		Genotype			
Maize	Environment	*Altiplano*	*Media*	*Huasteca*	Significance
	OTC	25.79 ± 1.75b			E × G (ns) E *** G ***
	Control	34.03 ± 1.79a			
	LSD	0.06			
		23.46 ± 1.36b	27.11 ± 1.42b	39.15 ± 2.61a	
	LSD	0.08			
Bean	OTC	28.86 ± 1.03c	29.9 ± 3.89c	52.11 ± 1.18a	E × G *** E ** G **
	Control	32.31 ± 0.51c	42.95 ± 0.92b	42.63 ± 0.71b	
	LSD	8.44			
Squash	OTC	29.96 ± 0.5c	39.98 ± 0.5b	63.89 ± 1.56a	E × G *** E *** G ***
	Control	32.62 ± 0.28c	40.96 ± 0.46b	43.12 ± 0.42b	
	LSD	3.63			
	Stomatal conductance (mol H_2_O m^−2^ s^−1^)				
Maize		Genotype			
		*Altiplano*	*Media*	*Huasteca*	E × G (ns) E *** G ***
	OTC	0.26 ± 0.01a			
	Control	0.15 ± 0.006b			
	LSD	0.03			
		0.18 ± 0.01b	0.2 ± 0.01b	0.25 ± 0.01a	
	LSD	0.04			
Bean	OTC	0.4 ± 0.03b	0.21 ± 0.02c	0.62 ± 0.02a	E × G ** E ** G *
	Control	0.22 ± 0.01c	0.22 ± 0.01c	0.55 ± 0.05ab	
	LSD	0.11			
Squash	OTC	0.52 ± 0.01a			E × G(ns) E (ns) G(ns)
	Control	0.49 ± 0.04a			
	LSD	0.09			
		0.5 ± 0.04a	0.54 ± 0.06a	0.47 ± 0.05a	
	LSD	0.13			
	Transpiration rates (mmol H_2_O m^−2^ s^−1^)				
Maize		Genotype			E × G (ns) E ** G **
		*Altiplano*	*Media*	*Huasteca*	
	OTC	4.11 ± 0.44a			
	Control	3.27 ± 0.33b			
	LSD	0.05			
		3.19 ± 0.17b	3.56 ± 0.16b	4.32 ± 0.23a	
	LSD		0.07		
Bean	OTC	6.1 ± 0.24b	4.92 ± 0.53b	9.27 ± 0.5a	E × G ** E ** G *
	Control	5.71 ± 0.28b	6.8 ± 0.59b	10.3 ± 0.36a	
	LSD	2.08			
Squash	OTC	7.81 ± 0.23a			E × G (ns) E (ns) G (ns)
	Control	7.25 ± 0.63a			
	LSD	0.21			
		6.82 ± 0.49a	7.75 ± 0.28a	8.02 ± 0.77a	
	LSD	0.31			
	Intrinsic water-use efficiency (µmol CO_2_ mol^−1^ H_2_O)				
		Genotype			
		*Altiplano*	*Media*	*Huasteca*	
	OTC	100.49 ± 11.6b			E × G(ns) E *** G(ns)
	Control	220.48 ± 18.55a			
	LSD	89.54			
		148.49 ± 12.97a	155.93 ± 13.78a	177.03 ± 15.9a	
	LSD	78.98			
Bean	OTC	75.56 ± 6.9c	143 ± 11.65b	85.25 ± 2.83c	E × G ** E ** G *
	Control	151.88 ± 11.52b	202.68 ± 13.43a	80.83 ± 6.74c	
	LSD	45.12			
Squash	OTC	61.57 ± 9.38b	69.89 ± 8.62b	169.16 ± 15.73a	E × G *** E *** G **
	Control	83.34 ± 13.13b	111.68 ± 20.09ab	88.73 ± 14.45b	
	LSD	66.49			

OTC: Open-top Chamber; E: Environment; G: Genotype; ns: no significant; * *t*-test, *p* < 0.05; ** *t*-test, *p* < 0.01, and *** *t*-test, *p* < 0.001. The letters a, b, c indicate significant differences according to the Tukey test (*p* < 0.05). The values are the means ± SE (standard error).

**Table 6:** Chlorophyll fluorescence parameters measured in different system of milpa from different environment under induced passive heat and controlled ambient at 75 days after emergence of each crop.

Electron Transport Rate (ETR) (μmol m^−2^ s^−1^)					
Genotype					
Crops	Environment	*Altiplano*	*Media*	*Huasteca*	Significance
Maize	OTC	47.8 ± 0.06b			E × G(ns) E *** G ***
	Control	61.42 ± 0.04a			
	LSD	4.65			
		61.6 ± 2.87a	49.05 ± 2.22b	53.19 ± 1.99b	
	LSD	6.84			
Bean	OTC	21.32 ± 2.52d	36.87 ± 2.76c	61.69 ± 0.45s	E × G * E * G ***
	Control	18.25 ± 1.51d	37.78 ± 1.58c	51.43 ± 0.89b	
	LSD	8.54			
Squash	OTC	50.33 ± 6.39b	50.33 ± 6.39b	50.33 ± 6.39b	E × G (ns) E ** G ***
	Control	82.19 ± 9.02a	82.19 ± 9.02a	82.19 ± 9.02a	
	LSD	13.5			
Maximum efficiency of the Photosystem II (Fv/Fm)					
Maize	Genotype				Significance
		*Altiplano*	*Media*	*Huasteca*	E × G (ns) E *** G(ns)
	OTC	0.75 ± 0.007b			
	Control	0.87 ± 0.001a			
	LSD	0.014			
		0.81 ± 0.01a	0.81 ± 0.01a	0.81 ± 0.01a	
	LSD	0.02			
Bean	OTC	0.76 ± 0.003bc	0.77 ± 0.004abc	0.74 ± 0.002c	E × G * E ** G(ns)
	Control	0.77 ± 0.016abc	0.78 ± 0.003ab	0.8 ± 0.002a	
	LSD		0.03		
Squash	OTC	0.76 ± 0.006a	0.75 ± 0.001a	0.75 ± 0.005a	E × G ** E ** G **
	Control	0.76 ± 0.007a	0.74 ± 0.002a	0.66 ± 0.02b	
	LSD	0.05			
	Quantum yield of the photosystem II (PhiPS2)				
Maize	Genotype				Significance
		*Altiplano*	*Media*	*Huasteca*	E × G(ns) E * G (ns)
	OTC	0.75 ± 0.007b			
	Control	0.87 ± 0.01a			
	LSD	0.014			
		0.81 ± 0.01a	0.81 ± 0.01a	0.82 ± 0.02a	
	LSD	0.03			
Bean	OTC	0.41 ± 0.09ab	0.38 ± 0.05ab	0.19 ± 0.009bc	E × G *** E * G **
	Control	0.5 ± 0.051a	0.08 ± 0.007c	0.35 ± 0.07ab	
	LSD	0.21			
Squash	OTC	0.58 ± 0.07ab	0.49 ± 0.05ab	0.37 ± 0.02bc	E × G ** E ** G ***
	Control	0.63 ± 0.02a	0.26 ± 0.04cd	0.16 ± 0.008d	
	LSD	0.15			
	Non-photochemical quenching (qN)				
Maize	Genotype				Significance
		*Altiplano*	*Media*	*Huasteca*	E × G(ns) E *** G(ns)
	OTC	0.82 ± 0.01b			
	Control	0.89 ± 0.007a			
	LSD	0.03			
		0.83 ± 0.01a	0.87 ± 0.008a	0.86 ± 0.02a	
	LSD	0.04			
Bean	OTC	0.52 ± 0.08c	0.61 ± 0.07bc	0.86 ± 0.006a	E × G ** E(ns) G(ns)
	Control	0.71 ± 0.02abc	0.8 ± 0.01ab	0.63 ± 0.04bc	
	LSD	0.23			
Squash	OTC		0.49 ± 0.06b		E × G(ns) E * G ***
	Control		0.65 ± 0.05a		
	LSD	0.11			
		0.33 ± 0.07b	0.62 ± 0.06a	0.77 ± 0.02a	
	LSD	0.16			
	Photochemical quenching (qP)				
Maize	Genotype				Significance
		*Altiplano*	*Media*	*Huasteca*	
	OTC	0.32 ± 0.002b	0.25 ± 0.008c	0.34 ± 0.001b	E × G *** E *** G ***
	Control	0.44 ± 0.008a	0.44 ± 0.008a	0.45 ± 0.008a	
	LSD	0.04			
Bean	OTC	0.59 ± 0.09b	0.69 ± 0.05ab	0.53 ± 0.01b	E × G *** E * G ***
	Control	0.71 ± 0.04ab	0.17 ± 0.007c	0.74 ± 0.04a	
	LSD	0.14			
Squash	OTC	0.83 ± 0.07ab	0.83 ± 0.03ab	0.71 ± 0.04bc	E × G ** E ** G ***
	Control	0.93 ± 0.003a	0.58 ± 0.06cd	0.4 ± 0.03d	
	LSD	0.21			
	Alternative non-photochemical quenching (NPQ)				
	Genotype				Significance
Maize		*Altiplano*	*Media*	*Huasteca*	E × G(ns) E * G(ns)
	OTC	1.36 ± 0.05b			
	Control	1.61 ± 0.06a			
	LSD	0.07			
		1.37 ± 0.007a	1.58 ± 0.06a	1.51 ± 0.08a	
	LSD	0.1			
Bean	OTC	0.89 ± 0.1c	1.006 ± 0.09c	1.44 ± 0.009c	E × G *** E(ns) G(ns)
	Control	1.09 ± 0.04abc	1.27 ± 0.02ab	0.9 ± 0.11c	
	LSD	0.36			
Squash	OTC	0.92 ± 0.15a			E × G(ns) E(ns) G ***
	Control	1.16 ± 0.13a			
	LSD	0.19			
		0.5 ± 0.14b	1.09 ± 0.13a	1.53 ± 0.11a	
	LSD	0.26			

OTC: Open-top chamber; LSD: Least Significant Difference; E: Environment; G: Genotype; ns: no significant; * *t*-test, *p* < 0.05; ** *t*-test, *p* < 0.01, and *** *t*-test, *p* < 0.001; The letters a, b, c, and d indicate significant differences according to the Tukey test (*p* < 0.05). The values are the means ± SE (standard error).

**Table 7:** Effect of induced passive heating on gas exchange parameters of the milpa system at 75 days after emergence from different climate of the state of San Luis Potosí (Mexico).

	Photosynthetic Rate (µmol CO_2_ m^−2^ s^−1^)				
Crops		Genotype			
	Environment	*Altiplano*	*Media*	*Huasteca*	Significance
Maize	OTC	50.9 ± 3.01a			E × G(ns) E(ns) G ***
	Control	55.37 ± 2.2a			
	LSD	0.06			
		40.44 ± 3.22b	57.69 ± 2.39a	61.28 ± 2.33a	
	LSD	0.09			
Bean	OTC	7.73 ± 0.91b			E × G(ns) E ** G **
	Control	14.29 ± 1.56a			
	LSD	0.26			
		4.58 ± 0.55b	8.57 ± 1.58ab	12.74 ± 1.85a	
	LSD	0.18			
Squash	OTC	9.67 ± 1.61b			E × G(ns) E * G ***
	Control	15.35 ± 2.11a			
	LSD	0.16			
		6.14 ± 1.24c	11.5 ± 2.29b	19.88 ± 1.76a	
	LSD	0.24			
	Stomatal conductance (mol H_2_O m^−2^ s^−1^)				
		Genotype			
Maize		*Altiplano*	*Media*	*Huasteca*	E × G(ns) E(ns) G(ns)
	OTC	0.22 ± 0.01a			
	Control	0.17 ± 0.01a			
	LSD	0.05			
		0.19 ± 0.02a	0.19 ± 0.01a	0.2 ± 0.01a	
	LSD		0.07		
Bean	OTC	0.34 ± 0.04a			E × G(ns) E(ns) G(ns)
	Control	0.29 ± 0.01a			
	LSD	0.08			
		0.28 ± 0.03a	0.38 ± 0.04a	0.31 ± 0.03a	
	LSD		0.12		
Squash	OTC	0.63 ± 0.04a			E × G(ns) E ** G(ns)
	Control	0.42 ± 0.03b			
	LSD	0.08			
		0.58 ± 0.05a	0.5 ± 0.06a	0.49 ± 0.04a	
	LSD	0.12			
	Transpiration rates (mmol H_2_O m^−2^ s^−1^)				
		Genotype			
		*Altiplano*	*Media*	*Huasteca*	
Maize	OTC	3.11 ± 0.69b	6.21 ± 0.57a	6.18 ± 0.46a	E × G * E * G *
	Control	4.46 ± 0.3ab	4.83 ± 0.45a	5.03 ± 0.35a	
	LSD	0.51			
Bean	OTC	3.67 ± 0.22b			E × G(ns) E * G **
	Control	4.67 ± 0.24a			
	LSD	0.07			
		4.13 ± 0.27ab	4.7 ± 0.34a	3.68 ± 0.26b	
	LSD	0.1			
Squash	OTC	0.63 ± 0.04a			E × G(ns) E ** G(ns)
	Control	0.42 ± 0.03b			
	LSD	0.08			
		0.58 ± 0.05a	0.5 ± 0.06a	0.49 ± 0.04a	
	LSD	0.13			
	Intrinsic water-use efficiency (µmol CO_2_ mol^−1^ H_2_O)				
Maize		Genotype			
		*Altiplano*	*Media*	*Huasteca*	
	OTC	286.83 ± 24.2b			E × G(ns) E * G(ns)
	Control	390.12 ± 38.4a			
	LSD	0.1			
		293.29 ± 38.06a	395.04 ± 44.4a	363.1 ± 37.98	
	LSD	0.15			
Bean	OTC	22 ± 4.47b			E × G(ns) E * G *
	Control	38.98 ± 5.38a			
	LSD	0.21			
		18.2 ± 2b	25.71 ± 5.86ab	47.56 ± 7.16a	
	LSD	0.3			
Squash	OTC	17.35 ± 3.54b			E × G(ns) E *** G ***
	Control	40.76 ± 6.21a			
	LSD	0.18			
		11.52 ± 2.34c	30.75 ± 8.4b	44.89 ± 5.15a	
	LSD	0.27			

OTC: Open-top Chamber; LSD: Least Significant Difference; E: Environment; G: Genotype; ns: no significant; * *t*-test, *p* < 0.05; ** *t*-test, *p* < 0.01, and *** *t*-test, *p* < 0.001. The letters a, b, c indicate significant differences according to the Tukey test (*p* < 0.05). The values are the means ± SE (standard error).

## Data Availability

Not applicable.

## Supplementary Materials

The following supporting information can be downloaded from: https://www.mdpi.com/article/10.3390/life12101589/s1. Table S1. Climatic characteristics of the three regions of the state of San Luis Potosí, Mexico. Table S2. Morphological and physiological variables and their description used to determine the effect of induced passive heat on milpa system from different climate of San Luis Potosí. Table S3. Results of the analysis of variance (ANOVA) of the morphological variables of the milpa system. Table S4. Results of the analysis of variance (ANOVA) of the yield and yield components variables of the milpa system. Table S5. Results of the analysis of variance (ANOVA) of the chlorophyll fluorescence parameters of the milpa system at 45 days after emergence. Table S6. Results of the Analysis of variance (ANOVA) of the gas exchange parameters of the milpa system at 45 days after emergence. Table S7. Results of the analysis of variance (ANOVA) of the chlorophyll fluorescence parameters of the milpa system at 75 days after emergence. Table S8. Results of the analysis of variance (ANOVA) of the gas exchange parameters of the milpa system at 75 days after emergence. Figure S1. Plots of the statistic correlation of Pearson linear (r) among the abiotic variables and various physiological parameters and yield of the milpa system.
