# Supplementary material for: Maize Intercropping in the Traditional “Milpa” System. Physiological, Morphological, and Agronomical Parameters under Induced Warming: Evidence of related Effect of Climate Change in San Luis Potosí (Mexico)
Source: Life (Basel). 2022 Oct 12;12(10):1589. doi: 10.3390/life12101589 (PMC9605515; doi:10.3390/life12101589)
Supplement: Supplementary file 1 [file life-12-01589-s001.zip › life-1915707-supplementary.pdf]

Supplementary Materials

Tables

Table S1. Climatic characteristics of the three regions of the state of San Luis Potosí, Mexico.

Table S2. Morphological and physiological variables and their description used to determine the effect of induced passive heat on *milpa* system from different climate of San Luis Potosí

Table S3. Results of the analysis of variance (ANOVA) of the morphological variables of the *milpa* system

Table S4. Results of the analysis of variance (ANOVA) of the yield and yield components variables of the *milpa* system

Table S5. Results of the analysis of variance (ANOVA) of the chlorophyll fluorescence parameters of the *milpa* system at 45 days after emergence.

Table S6. Results of the Analysis of variance (ANOVA) of the gas exchange parameters of the *milpa* system at 45 days after emergence

Table S7. Results of the analysis of variance (ANOVA) of the chlorophyll fluorescence parameters of the *milpa* system at 75 days after emergence.

Table S8. Results of the analysis of variance (ANOVA) of the gas exchange parameters of the *milpa* system at 75 days after emergence.

Figures

Figure S1. Plots of the statistic correlation of Pearson linear (r) among the abiotic variables and various physiological parameters and yield of the *milpa* system. AHU: accumulated heat units; Tmean: mean daily temperature; Tmax: Maximum daily temperature; Tmin: minimum daily temperature; P1: at 15 cm and P2: at 150 cm above the soil; ETR: Electron transport rate; Fv/Fm: Maximum PSII efficiency; PhiPS2: quantum yield of PSII; qN: Non-photochemical quenching; NPQ: Alternative non-photochemical quenching; qP: Photochemical quenching; Photo: photosynthetic rate; Cond: stomatal conductance; Trmmol: transpiration rates; iWUE: intrinsic water use efficiency; 1 and 2: measured at 45 and 75 days after emergence, respectively. The boxed plots are significant at $p < 0.05$.

Table S1. Climatic characteristics of the three regions of the state of San Luis Potosí, Mexico.

Milpa	Region	Geographic coordinates of the points of collection	Predominate climate based on modifications of the Köppen climate classification system	Climate
**Maize+Bean+ Squash**	*Altiplano*	2 062 m a.s.l.<br>-101°7′W 22°16N	BS1kw(e)gw"	warm-dry
	Media	1 390 m a.s.l.<br>-99°32′W 22°8′N	Cb(w2)(w)(I′)	temperate
	Huasteca	225 m a.s.l.<br>-98°58′W 21°35′N	Am(e)gw"	hot and humid
**Reference**		García, 2004.		

Table S2. Morphological and physiological variables and their description used to determine the effect of induced passive heat on *milpa* system from different climate of San Luis Potosí

Variables	Maize	Bean	Squash
**Leaf number per plant**	10 competitive plants were selected and counted the number of leaves in each plot by environment (control and OTC).	5 competitive plants were selected and counted the number of leaves in each plot by environment (control and OTC).	3 competitive plants were selected and counted the number of leaves in each plot by environment (control and OTC).
**Leaf length (cm)**	10 leaves were selected just after the ear insertion.		
**Width leaf (cm)**	10 leaves were selected just after the ear insertion.		
**Leaf area (cm^2)**	Were calculated with data of the length and width.		
**Rate of growth**	10 competitive plants were selected and determined in a period of 135 days.	5 competitive plants were selected and determined in a period of 105 days.	3 competitive plants were selected and determined in a period of 105 days.
**Height to ear insertion (m)**	10 competitive plants were selected and determined in each plot.		
**Stem thickness (mm)**	10 competitive plants were selected and determined in each plot.	5 competitive plants were selected and determined in each plot.	3 competitive plants were selected and determined in each plot.
**Plant height**	10 competitive plants were selected and determined in each plot.	5 competitive plants were selected and determined in each plot.	3 competitive plants were selected and determined in each plot.
**Days for female flowering per plot**	In each plot, the female flowering in days to 50% silking.		
**Days for male flowering per plot**	In each plot, the male flowering in days to 50% anther extrusion.		
**Number of flowers per plant**		5 competitive plants were selected and counted the number of flowers in each plot.	3 competitive plants were selected and counted the number of flowers in each plot.
**Chlorophyll fluorescence parameters**	6 competitive plants in each were selected and measured from 13h to 16h in two moments.	3 competitive plants in each were selected and measured from 13h to 16h in two moments.	3 competitive plants in each were selected and measured from 13h to 16h in two moments.
**Gas exchange parameters**	6 competitive plants in each were selected and measured from 9h to 11h in two moments.	3 competitive plants in each were selected and measured from 9h to 11h in two moments.	3 competitive plants in each were selected and measured from 9h to 11h in two moments.

Table S3. Results of the analysis of variance (ANOVA) of the morphological variables of the *milpa* system

**Factor of variation for every crop**	PH		LNP		LL		WL		RG		LA	
**Maize**	CM	Fvalue	CM	Fvalue	CM	Fvalue	CM	Fvalue	CM	Fvalue	CM	Fvalue
E	7.88	115.1***	27.07	12.8**	72.6	0.99ns	134.9	151.3***	0.005	47.7***	527764.5	61.31***
G	2.84	41.4***	108.16	51.3***	2046.5	27.8***	9.7	10.89***	0.005	42.4***	231974.9	26.95***
E x G	0.31	4.55**	5.5	2.6ns	44.9	0.61ns	8.28	9.29**	0.0005	4.55**	21520.4	2.5ns
CV(%)	11.3		11.9		8.9		9.34		9.8		12.7	
	DFF		DMF		HEI		ST					
	CM	Fvalue	CM	Fvalue	CM	Fvalue	CM	Fvalue				
E	60.7	3.1ns	114.08	11.9**	1.17	106.5***	1.19	0.1ns				
G	184.3	9.41**	102.6	10.7**	0.05	5.03**	104.07	8.45**				
E x G	4	0.2ns	28.08	2.93ns	0.02	1.67ns	64.87	5.27**				
CV(%)	7.4		5.1		9.7		10.75					
	NFP		NLP		PH		RG		ST			
**Bean**	CM	Fvalue	CM	Fvalue	CM	Fvalue	CM	Fvalue	CM	Fvalue		
E	1215	116.83***	2856.6	39.03***	24.77	1.13ns	0.04	11.54**	0.05	0.03ns		
G	38.06	3.66*	2027.92	27.71***	77.13	3.52*	0.01	3.89*	102.29	67.76***		

E x G	8.6	0.83ns	453.65	6.2**	1.23	0.06ns	0.0083	2.32ns	6.43	4.26**		
CV(%)	19		25.19		12.53		16.31		16.31			
	NFP		NLP		PH		RG		ST			
**Squash**	CM	Fvalue	CM	Fvalue	CM	Fvalue	CM	Fvalue	CM	Fvalue		
E	1.94	40.07***	5675.11	142.39***	696.96	19.97**	0.09	36.8***	266.88	14.81**		
G	0.16	3.25ns	425.36	10.67**	204.95	5.87**	0.05	20.94***	21.67	1.2ns		
E x G	0.03	0.67ns	200.19	5.02**	187.12	5.36**	0.05	20.94***	79.86	4.43*		
CV(%)	25.52		13.17		5.77		7.68		18.74			

PH: plant height; LNP: leaf number per plant; LL: leaf length; WL: width leaf; RG: rate of growth; LA: leaf area; DFF: days to female flowering; DMF: days to male flowering; HEI: height to ear insertion; ST: stem thickness; NFP: number of flowers per plant; NLP: number of leaves per plant; PH: plant height; NFP: number of flowers per plant; NLP: number of leaves per plant; CV: coefficient of variation; ns: no significant; * t-test, $p < 0.05$; ** t-test, $p < 0.01$, and *** t-test, $p < 0.001$.

Table S4. Results of the analysis of variance (ANOVA) of the yield and yield components variables of the *milpa* system

	NCP		CL		CD		CW		NRPC		NGPR	
**Maize**	CM	Fvalue	CM	Fvalue	CM	Fvalue	CM	Fvalue	CM	Fvalue	CM	Fvalue
E	3.84	103.6***	468.07	145.17***	305.6	26.27***	0.47	35.72***	4.4	2.75ns	1442.13	38.64***
G	0.16	4.37**	1.98	0.62ns	156.3	13.44***	0.3	22.95***	19.82	12.38***	217.2	5.82**
E x G	0.07	2.02ns	3.43	1.07ns	477.5	41.05***	0.09	6.91**	7.75	4.84**	35.27	0.95ns
CV(%)	14.93		10.99		6.48		5		11.9			
	100GW		YIELD									
	CM	Fvalue	CM	Fvalue								
E	137.58	15.43**	0.2	30.05**								
G	138.21	15.5**	0.08	12.14**								
E x G	25.5	2.87ns	0.02	2.61ns								
CV(%)	6.5		14.19									

	YIELD	
**Bean**	CM	Fvalue
E	0.07	26.73**
G	0.47	168.37**
E x G	0.03	11.43**
CV(%)	8.33	

	YIELD	
**Squash**	CM	Fvalue
E	1.93	114.89***
G	0.2	11.95***
E x G	0.03	2.37ns
CV(%)	8.16	

NCP: number of cob per plant; CL: cob length; cd: cob diameter; CW: cob weight; NRPC: number of rows per cob; NGPR: number of grains per row; 100GW: 100 grains weight for maize; CV: coefficient of variation; ns: no significant; * t-test, $p < 0.05$;** t-test, $p < 0.01$, and *** t-test, $p < 0.001$.

Table S5. Results of the analysis of variance (ANOVA) of the chlorophyll fluorescence parameters of the *milpa* system at 45 days after emergence.

	**ETR**		**Fv/Fm**		**NPQ**		**PhiPS2**		**qN**		**qP**	
**Maize**	CM	Fvalue	CM	Fvalue	CM	Fvalue	CM	Fvalue	CM	Fvalue	CM	Fvalue
Environment (E)	0.02	1.2ns	0.01	9.4**	0.23	15.63***	0.05	25.5***	0.09	29.9***	0.002	0.3ns
Genotypes(G)	0.11	6.97**	0.005	3.05ns	0.09	5.96**	0.01	5.63**	0.01	3.52*	0.02	4.41*
E x G	0.04	2.71ns	0.0002	0.13ns	0.03	2.01ns	0.004	2.07ns	0.008	2.9ns	0.001	0.29ns
CV	7.32		5.5		9.57		17.09		6.32		13.64	
**Bean**												
Environment (E)	142.02	9.6*	0.05	13.03**	0.01	1.86ns	0.04	110.05***	0.12	243.7***	0.0004	0.04ns
Genotypes(G)	3351.6	227.2***	0.0003	0.09ns	0.37	47.4***	0.13	303.4***	0.09	197.03***	0.0007	0.07ns
E x G	90.6	6.15**	0.007	2ns	0.72	91.6***	0.004	10.6**	0.03	63.8***	0.09	9.12**
CV	13.7		9.88		9.27		5.98		6.01		16.04	
**Squash**												
Environment (E)	0.26	13.7**	0.02	4.69*	0.12	2.17ns	0.007	0.62ns	0.03	3.11ns	0.11	7.22*
Genotypes(G)	0.3	15.9***	0.004	1.06ns	0.76	13.6***	0.02	2.42ns	0.18	17.2***	0.05	3.48*
E x G	0.57	30.5***	0.01	2.35ns	0.15	2.76ns	0.04	3.84*	0.17	16.1***	0.12	7.77**
CV	8.78		12.6		28.01		19.12		17.48		18.32	

ETR: Electron transport rate; Fv/Fm: Maximum PSII efficiency; PhiPS2: quantum yield of PSII; qN: non-photochemical quenching; NPQ: Alternative non-photochemical quenching; qP: Photochemical quenching; OTC: Open top chamber; LSD: Least Significant Difference; ns: no significant; * t-test, $p < 0.05$;** t-test, $p < 0.01$, and *** t-test, $p < 0.001$.

Table S6. Results of the Analysis of variance (ANOVA) of the gas exchange parameters of the *milpa* system at 45 days after emergence

	Cond		iWUE		Photo		Trmmol	
**Maize**	CM	Fvalue	CM	Fvalue	CM	Fvalue	CM	Fvalue
Environment (E)	0.22	58.42***	2.18	198.8***	0.32	19.8***	0.16	14.43**
Genotypes(G)	0.03	9.66**	0.03	2.99*	0.29	18.04***	0.1	9.1**
E x G	0.006	1.67ns	0.0007	0.07ns	0.01	0.61ns	0.005	0.46ns
CV	13.93		4.84		8.89		19.54	
**Bean**								
Environment (E)	0.04	9.6*	0.18	23.7***	0.02	4.2*	0.11	2.31ns
Genotypes(G)	0.28	66.7***	0.29	37.54***	0.11	23.09***	2.21	43***
E x G	0.02	5.53**	0.08	11.2**	0.05	11.03**	0.28	5.45**
CV	11.09		4.35		4.46		8.62	
**Squash**								
Environment (E)	0.008	0.49ns	0.00004	0ns	0.016	48.06***	0.13	1.39ns
Genotypes(G)	0.004	0.23ns	0.17	5.99**	0.15	439.54***	0.16	1.68ns
E x G	0.04	2.57ns	0.2	6.86**	0.03	107.67***	0.19	2.06ns
CV	19.07		8.95		1.15		11.43	

Photo: photosynthetic rate; Cond: stomatal conductance; Trmmol: transpiration rates; iWUE: intrinsic water use efficiency; ns: no significant; * t-test, $p < 0.05$;** t-test, $p < 0.01$, and *** t-test, $p < 0.001$.

Table S7. Results of the analysis of variance (ANOVA) of the chlorophyll fluorescence parameters of the *milpa* system at 75 days after emergence.

**Maize**	ETR		Fv/Fm		NPQ		PhiPS2		qN		qP	
	CM	Fvalue	CM	Fvalue	CM	Fvalue	CM	Fvalue	CM	Fvalue	CM	Fvalue
Environment (E)	0.19	42.3***	0.27	310.9***	0.19	9.16*	0.04	6.04*	0.07	21.4***	0.24	375.9***
Genotypes(G)	0.05	13.19***	0.0006	0.74ns	0.05	2.38ns	0.005	0.85ns	0.01	2.8ns	0.01	19.24***
E x G	0.0004	0.1ns	0.0003	0.4ns	0.03	1.74ns	0.007	1.1ns	0.005	1.52ns	0.008	13.15***
CV	3.9		3.67		12.13		27.8		7.01		4.24	
**Bean**												
Environment (E)	154.1	6.51*	0.0039	11.3**	0.006	0.15ns	0.009	0.66ns	0.02	1.34ns	0.03	4.74*
Genotypes(G)	4058.6	171.3***	0.0003	0.89ns	0.11	2.62ns	0.13	9.28**	0.04	3.01ns	0.13	20.55***
E x G	96.15	4.06*	0.002	6.34*	0.61	14.6***	0.19	13.05***	0.18	10.96**	0.26	40.4***
CV	12.8		2.41		18.58		22.8		18.68		10.96	
**Squash**												
Environment (E)	0.42	12.5**	0.01	15.47**	0.19	2.56ns	0.12	16.68**	0.11	4.29*	0.21	15.37**

Genotype(G)	0.67	19.67***	0.009	12.56**	1.16	15.39***	0.22	29.13***	0.39	14.85***	0.31	22.52***
G x E	0.06	1.86ns	0.006	8.78**	0.09	1.2ns	0.06	8.24**	0.02	0.83ns	0.14	10.2**
CV	10.6		3.64		29.02		14.09		22.45		16.5	

ETR: Electron transport rate; Fv/Fm: Maximum PSII efficiency; PhiPS2: quantum yield of PSII; qN: non-photochemical quenching; NPQ: Alternative non-photochemical quenching; qP: Photochemical quenching; OTC: Open top chamber; LSD: Least Significant Difference; ns: no significant; * t-test, $p < 0.05$;** t-test, $p < 0.01$, and *** t-test, $p < 0.001$.

Table S8. Results of the analysis of variance (ANOVA) of the gas exchange parameters of the *milpa* system at 75 days after emergence.

**Maize**	Cond		iWUE		Photo		Trmmol	
	CM	Fvalue	CM	Fvalue	CM	Fvalue	CM	Fvalue
Environment (E)	0.03	2.45ns	0.22	4.83*	0.07	3.65ns	0.008	0.04ns
Genotypes (G)	0.003	0.25ns	0.11	2.4ns	0.33	16.82***	1.74	9.64**
G x E	0.045	3.7ns	0.11	2.4ns	0.05	2.76ns	1.08	6*
CV	25.7		8.81		8.34		19.57	
**Bean**								
Environment (E)	0.02	1.36ns	0.82	8.99*	0.58	8.34**	0.1	9.8**
Genotypes (G)	0.02	1.64ns	0.41	4.57*	0.5	7.27**	0.03	3.1*
G x E	0.025	1.68ns	0.045	0.49ns	0.1	1.49ns	0.01	1.72ns
CV	21.89		22.38		32.25		17.3	
**Squash**								
Environment (E)	0.2	13.95**	1.58	22.01***	0.48	8.22*	0.24	14.69**
Genotypes (G)	0.01	0.86ns	1.2	16.69***	0.98	16.81***	0.005	0.33ns
G x E	0.01	0.82ns	0.02	0.3ns	0.036	0.63ns	0.01	0.69ns
CV	16.81		20.83		24.79		21.1	

Photo: photosynthetic rate; Cond: stomatal conductance; Trmmol: transpiration rates; iWUE: intrinsic water use efficiency; ns: no significant; * t-test, $p < 0.05$;** t-test, $p < 0.01$, and *** t-test, $p < 0.001$.

Figure S1. Plots of the statistic correlation of Pearson linear (r) among the abiotic variables and various physiological parameters and yield of the *milpa* system.

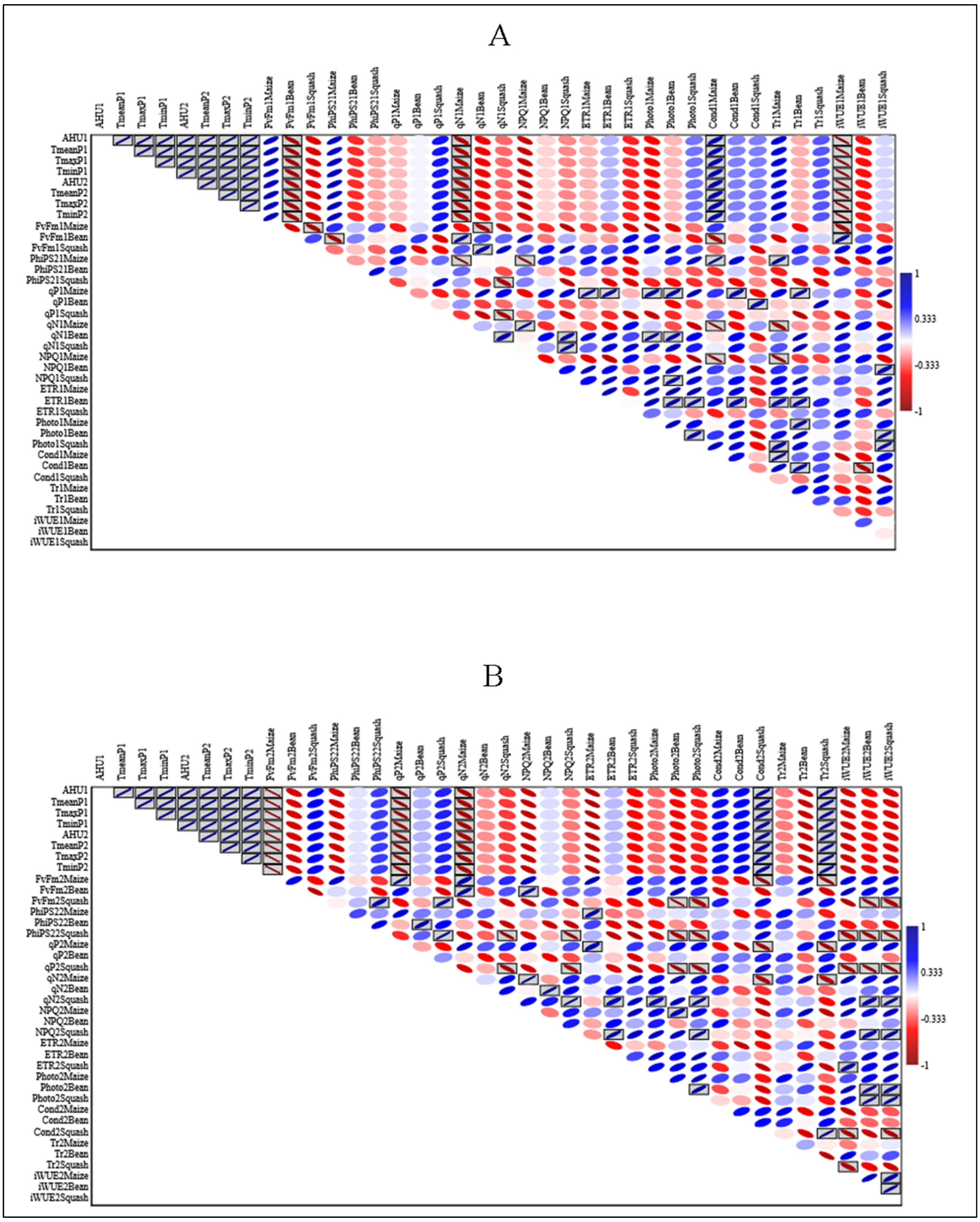

AHU: accumulated heat units; Tmean: mean daily temperature; Tmax: Maximum daily temperature; Tmin: minimum daily temperature; P1: at 15 cm and P2: at 150 cm above the soil; ETR: Electron transport rate; Fv/Fm: Maximum PSII efficiency; PhiPS2: quantum yield of PSII; qN: Non-photochemical quenching; NPQ: Alternative non-photochemical quenching; qP: Photochemical quenching; Photo: photosynthetic rate; Cond: stomatal conductance; Trmmol: transpiration rates; iWUE: intrinsic water use efficiency; 1 and 2: measured at 45 and 75 days after emergence, respectively. The boxed plots are significant at $p < 0.05$.
